# Copper–phenanthroline catalysts for regioselective synthesis of pyrrolo[3′,4′:3,4]pyrrolo[1,2-*a*]furoquinolines/phenanthrolines and of pyrrolo[1,2-*a*]phenanthrolines under mild conditions

**DOI:** 10.3762/bjoc.10.62

**Published:** 2014-03-20

**Authors:** Rupankar Paira, Tarique Anwar, Maitreyee Banerjee, Yogesh P Bharitkar, Shyamal Mondal, Sandip Kundu, Abhijit Hazra, Prakas R Maulik, Nirup B Mondal

**Affiliations:** 1 Department of Chemistry, Indian Institute of Chemical Biology, Council of Scientific and Industrial Research, 4 Raja S.C. Mullick Road, Jadavpur, Kolkata-700032, India

**Keywords:** copper(II) chloride–phenanthroline, 1,3-dipolar cycloaddition, furo[3,2-*h*]quinoliniums, phenanthroliums, pyrrolo[1,2-*a*]phenanthrolines, pyrrolo[3′,4′:3,4]pyrrolo[1,2-*a*]furoquinolines/phenanthrolines

## Abstract

A new series of pyrrolo[3′,4′:3,4]pyrrolo[1,2-*a*]furoquinolines/phenanthrolines and pyrrolo[1,2-*a*]phenanthrolines were efficiently built up from an 8-hydroxyquinoline derivative or phenanthroline via 1,3-dipolar cycloaddition reaction involving non-stabilized azomethine ylides, generated in situ from the parent furo[3,2-*h*]quinoliniums/phenanthroliums, in presence of a copper(II) chloride–phenanthroline catalytic system. The methodology combines general applicability with high yields.

## Introduction

The chemistry of the 1,3-dipolar cycloaddition has always been a fascinating undertaking especially when azomethine ylides are involved as the key component [1–9]. These ylides, both stabilized or non-stabilized 1,3-dipoles, can easily enter the reaction independent of their stability and lead to the formation of a pyrrolidine core, a structural motif of immense interest from both chemical and pharmacological points of view [10]. In recent years, there have been many attempts to synthesize diversely modified pyrrolidines, both symmetric and asymmetric in nature. Several of these attempts involved 1,3-dipolar cycloaddition reactions involving azomethine ylides to give cycloadducts, which were further explored as potential antiviral, antifungal, antitumor and anti-HIV candidates [1–15]. We have also been pursuing the cycloaddition methodology for several years and established some synthetic routes towards indolizines, pyrrolo[1,2-*a*]quinolines/isoquinolines, oxazadicyclopenta[*a*,*h*]naphthalenes etc., some of which have been evaluated as potential antibacterial and antifungal agents [16–19]. In order to explore the possibility of using structurally more complex dipoles and dipolarophiles to construct more interesting structural networks, we replaced simple alkenes/alkynes with maleimide derivatives. Our preference for this dienophile was dictated by a recent patent on the lifespan altering properties of cycloadducts involving maleimide dienophiles [20]. Moreover, very recently we have characterized some furo[3,2-*h*]quinoliniums as potent non-detergent spermicides [19], which encouraged us for further modification and derivatization of furoquinoline analogues in search for more potent agents. Thus we became interested in the construction of a number of structurally complex pentacyclic pyrrolo[3′,4′:3,4]pyrrolo[1,2-*a*]furoquinolines, for possible identification of new antibacterial/antifungal/spermicidal/lifespan altering agents, where furo[3,2-*h*]quinoliniums were employed as dipole precursors and maleimide derivatives as dipolarophiles.

We initially employed the protocols from our recently developed green methodologies [21–25], which however failed to give any promising outcome and forced us to explore new catalytic systems. While searching for this goal, we were attracted by the possible application of copper-catalysis, which has always been an effective tool especially with Diels–Alder reactions [26–27]. Thus, we studied the effect of a number of catalytic systems and after an extensive screening, we found copper(II) chloride–phenanthroline as the best catalytic pair for this purpose. Herein we wish to present the results of our recent synthetic efforts to synthesize a series of unique pentacyclic pyrrolo[3′,4′:3,4]pyrrolo[1,2-*a*]furoquinolines/phenanthrolines using the above catalytic system. To the best of our knowledge, this is the first report of the application of this catalyst for the regioselective 1,3-dipolar cycloaddition reaction, involving azomethine ylides derived from structurally complex quinoline-based N-heterocycles.

## Results and Discussion

**Scheme 1:**
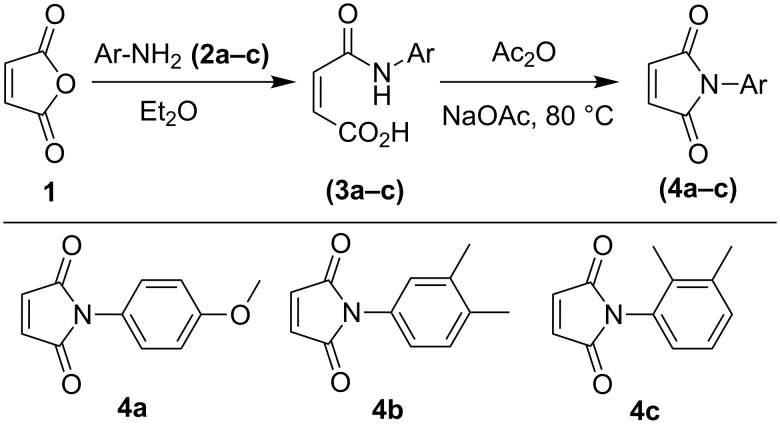
Preparation of maleimide dipolarophiles **4a**–**c**.

**Scheme 2:**
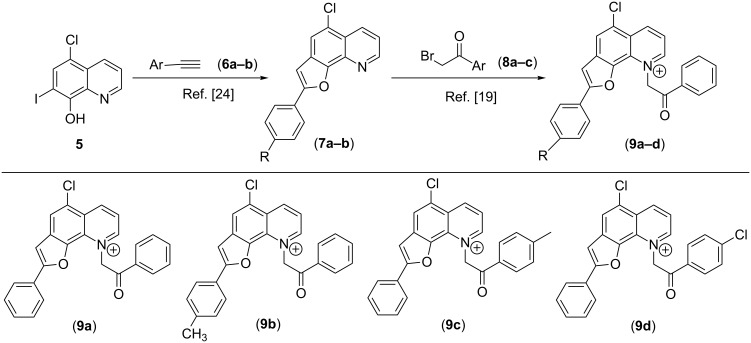
Preparation of 1,3-dipole precursors **9a–d**.

Our studies started with the preparation (Scheme 1 and Scheme 2) of maleimides **4a**–**c** employing maleic anhydride (**1**) and different aromatic amines **2a**–**c,** and of furo[3,2-*h*]quinoliniums **9a**–**d** from 5-chloro-8-hydroxy-7-iodoquinoline **5** [19,24]. However, attempted condensation of **4a** with the 1,3-dipole generated in situ from **9a**, employing protocols from our recently developed methodologies, did not succeed, affording products only in low yields (10–19%, Table 1, entries 1–7).

**Table 1:** Optimization of conditions of reaction between **4a** and **9a**.

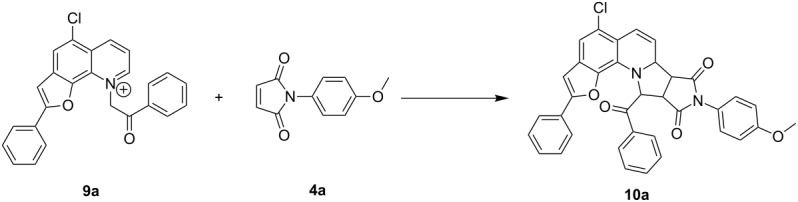					

Entry	Catalytic system	Solvent^a^	Time (h)	Temp. (°C)	Yield^b^

1	Basic alumina	None	0.3	80	10^c^
2	Amberlite IRA 402 (OH)	H_2_O	10	90	16
3	K-10 clay	None	0.3	80	12^c^
4	Triton X-114 (60 mM)	H_2_O	3	rt	17
5	SDS (60 mM)	H_2_O	3	rt	15
6	TTAB (80 mM)	H_2_O	6	rt	19
7	CTAB (90 mM)	H_2_O	5	rt	12
8	SnCl_4_	Toluene	8	80	NR
9	Sc(OTf)_3_	DCM	10	rt	NR
10	Sc(OTf)_3_	Toluene	10	80	NR
11	Mg(ClO_4_)_3_	DCM	10	rt	NR
12	Mg(ClO_4_)_3_	Toluene	10	80	11
13	Cu(OTf)_2_	DCM	8	rt	15
14	Cu(OTf)_2_	Toluene	8	80	28
15	Cu(OAc)_2_	DCM	8	rt	12
16	Cu(OAc)_2_	Toluene	8	80	32
17	Cu(OAc)_2_	MeCN	8	65	47
18	CuCl_2_	DCM	6	rt	23
19	CuCl_2_	Toluene	6	80	45
20	CuCl_2_	MeCN	3	65	57
21	CuCl_2_/PPh_3_	MeCN	3	65	58
22	CuCl_2_/PMePh_2_	MeCN	3	65	58
23	CuCl_2_/PCy_3_	MeCN	3	65	56
24	CuCl_2_/P(3-ClC_6_H_4_)_3_	MeCN	3	65	58
25	CuCl_2_/P(3-OMeC_6_H_4_)_3_	MeCN	3	65	55
26	CuCl_2_/DPEphos	MeCN	3	65	65
27	CuCl_2_/xantphos	MeCN	3	65	67
28	CuCl_2_/pyphos	MeCN	3	65	65
29	CuCl_2_/bis-oxazocine	MeCN	3	65	74
30	CuCl_2_/pyrimidine	MeCN	3	65	71
31	CuCl_2_/phenanthroline (**L_1_**)	MeCN	3	65	94
32	CuCl_2_/phenanthroline (**L_2_**)	MeCN	3	65	94

^a^All the reactions were performed in presence of DBU; ^b^Isolated yield; ^c^The reactions were performed under microwave irradiation at 180 W.

**Figure 1:**
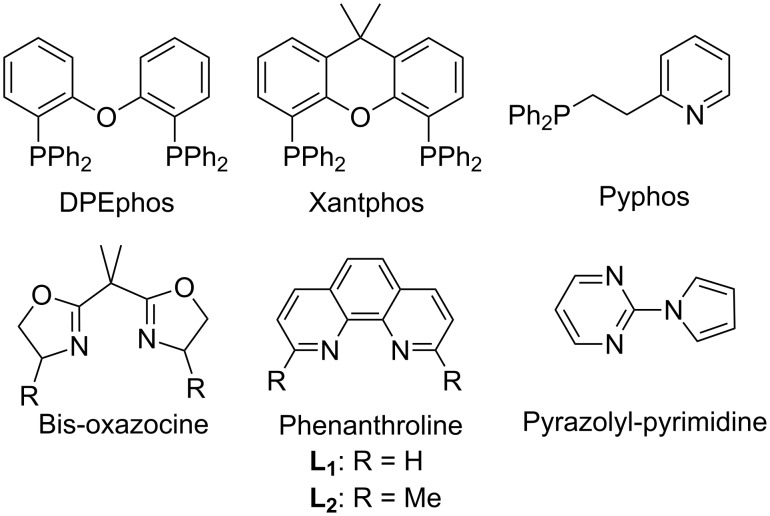
Bi-/tridentate ligands used for the optimization of the reaction conditions.

We then studied the feasibility of a metal-catalyzed 1,3-dipolar cycloaddition strategy. A thorough screening of different catalysts, as summarized in Table 1, revealed the supremacy of copper catalysts in this particular reaction over the others; CuCl_2_ appeared to be the catalyst of choice (Table 1, entries 8–32). In order to explore the effect of ligands, a number of phosphines, bis-oxazocines, pyrazolyl-pyrimidines and phenanthroline analogues were employed (Figure 1). As represented in Table 1, the monodentate ligands are in general less effective (55–58% yield; Table 1, entries 21–25) than bi-/tridentate ligands (65–94% yield; Table 1, entries 26–32), and phosphines in general proved less effective in terms of product yield (Table 1, entries 21–28). Bis-oxazocines and pyrazolyl-pyrimidines on the other hand showed some promising results (Table 1, entries 29 and 30). However, both in terms of yield and cleaner reaction profile, 1,10-phenanthrolines (**L_1_**, **L_2_**) were identified as the best partners for CuCl_2_ (Table 1, entries 31 and 32). Thus, the 1,3-dipolar cycloaddition reaction between **9a** (1 equiv) and **4a** (1.1 equiv) yielded 94% of **10a** within 3 h, when 5 mol % CuCl_2_ and 5 mol % of either **L_1_** or **L_2_** were employed with DBU as the base in acetonitrile solvent at 65 °C. This was taken as the best conditions to perform the reaction.

**Figure 2:**
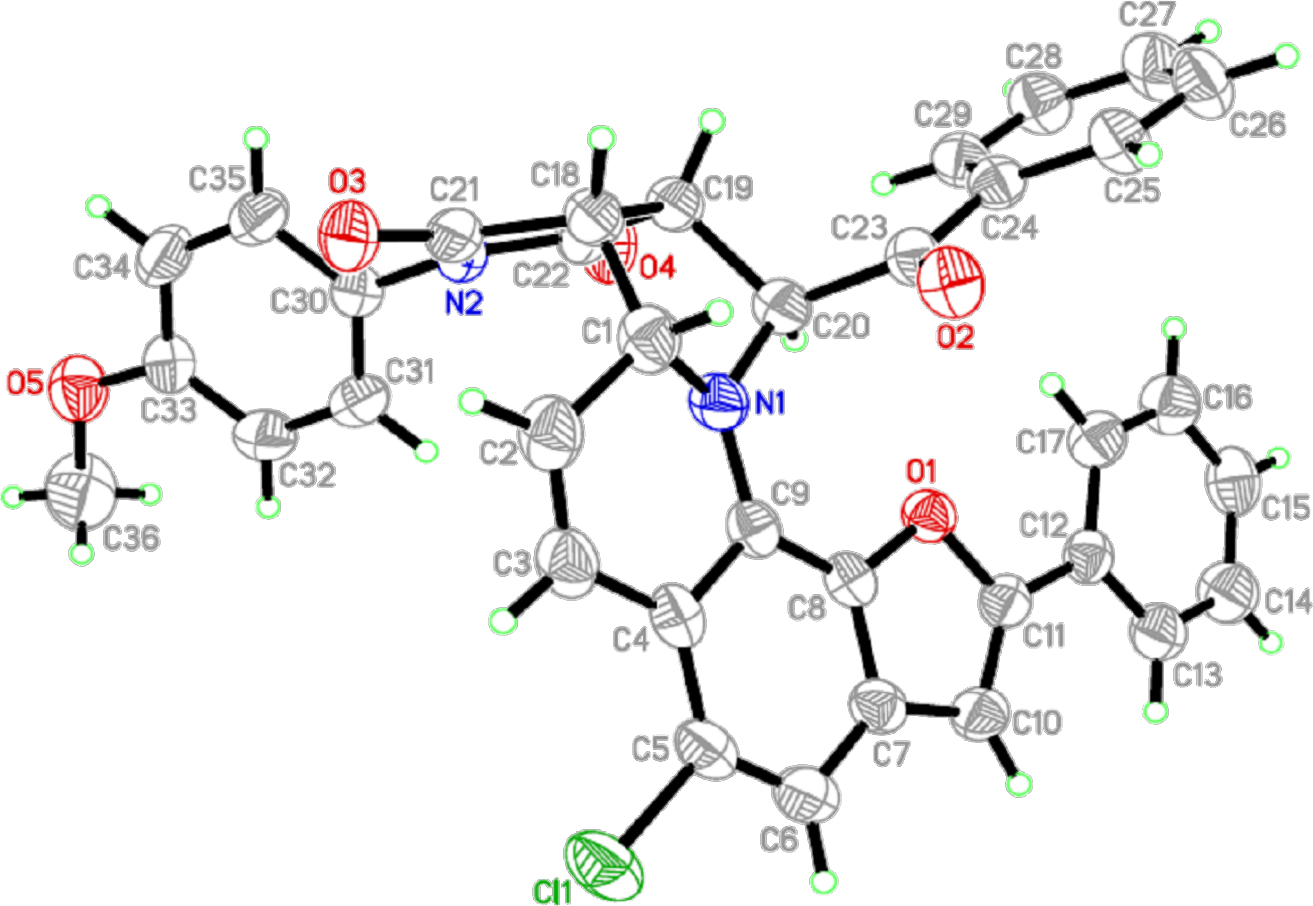
ORTEP diagram showing the molecular structure of **10a** at 30% probability level.

The structure of **10a** was deduced from the appearance of a new doublet of doublets at δ 5.50 and δ 6.20. Another doublet at δ 3.90 and a new multiplet at δ 3.50 also indicated the success of the reaction, as these signals could be attributed to the new pyrrolidine core. Besides, the cluster in the aromatic region (δ 6.60–8.50) indicated the presence of 19 aromatic protons in the cycloadduct **10a**. This interpretation was also well supported by the ^13^C NMR of **10a**. Peaks for the four *sp*^3^-methine carbons of the newly formed pyrrolidine motif appeared at δ 47.2, 47.4, 61.0 and 66.5 as expected. Two methine carbon signals appearing at δ 101.6 and 110.8 could be assigned to the CH units present in the dihydroquinoline ring. Moreover, the presence of peaks for three carbonyl carbons at δ 174.9, 176.6, 194.5, and for ten other quaternary carbons and 19 methine carbons in the aromatic region (δ 116.0–159.8) also confirmed the structure. The relative orientations of H-1, H-18, H-19 and H-20 could be determined unambiguously by the single-crystal X-ray diffraction analysis of **10a**; the ORTEP diagram is presented in Figure 2. No other diastereomer could be detected.

**Scheme 3:**
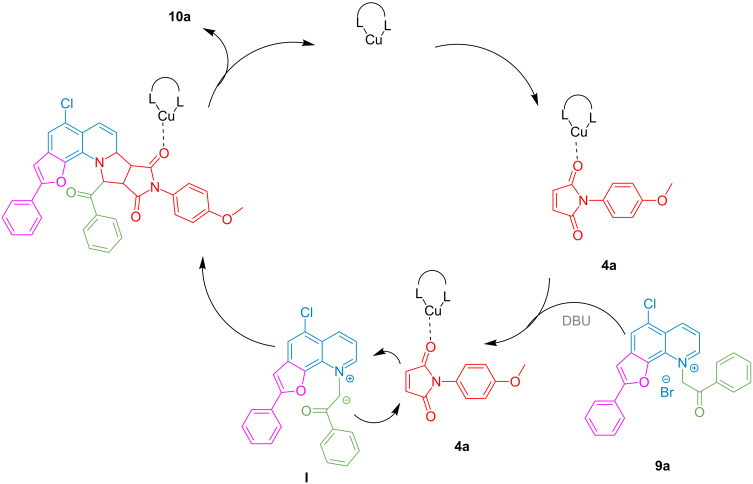
Plausible mechanistic pathway for the synthesis of pyrrolo[3′,4′:3,4]pyrrolo[1,2-*a*]furoquinolines.

The plausible mechanism of this cycloaddition is presented in Scheme 3. The base (DBU) abstracts the acidic proton of furo[3,2-*h*]quinolinium **9a** to generate the 1,3-dipole **I**. The Cu(II)–phenanthroline system activates the maleimide dipolarophile via coordination with the carbonyl group to undergo a [3 + 2] cycloaddition with the 1,3-dipole to form the cycloadduct **10a**, releasing the Cu(II) complex to enter another cycle.

**Scheme 4:**
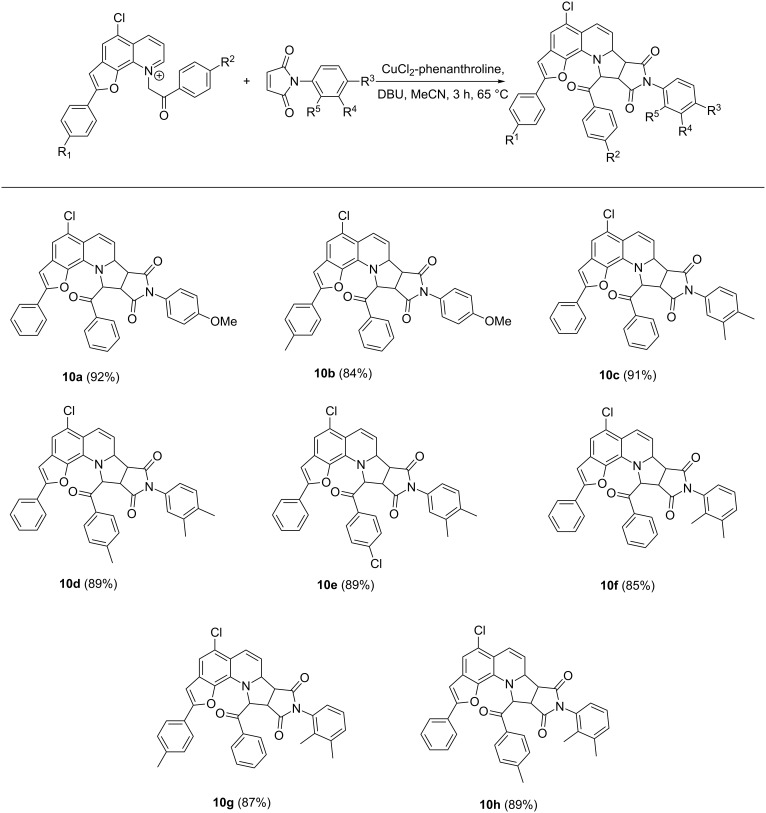
Synthesis of pyrrolo[3′,4′:3,4]pyrrolo[1,2-*a*]furoquinoline analogues under the optimized protocol.

In order to establish the general applicability of this protocol, we reacted different furo[3,2-*h*]quinoliniums **9a**–**d** and maleimide dipolarophiles **4a**–**c** under the standardized reaction conditions. As obvious from the results summarized in Scheme 4, all the reactions proceeded smoothly to give cycloadducts with excellent yields, which were fully characterized by mass and NMR analysis.

**Scheme 5:**
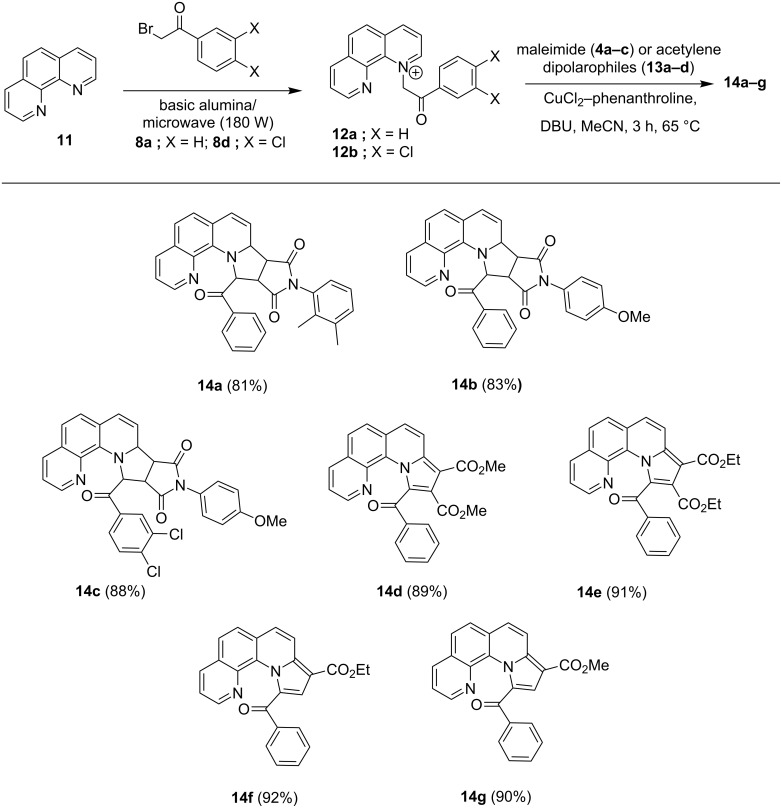
Construction of pyrrolo[3′,4′:3,4]pyrrolo[1,2-*a*]phenanthrolines **14a**–**c** and of pyrrolo[1,2-*a*]phenanthrolines **14d**–**g**.

In order to test its general applicability further, we replaced furo[3,2-*h*]quinoliniums with phenanthroliniums **12a,b**. These phenanthroliniums were synthesized (Scheme 5) from phenanthroline (**11**) and 2′-bromoacetophenones **8a** and **8d**, under basic alumina/microwave (180 W) conditions. These were then subjected to the optimized [3 + 2] cycloaddition protocol with different *N*-phenylmaleimide dipolarophiles **4a**–**c**, which eventually produced similar cycloadducts **14a**–**c** in high yield. It is interesting to note that every reaction proceeded only up to the cycloaddition stage, without any aromatization of the final cycloadduct. However, a similar reaction of phenanthroliniums **12a**,**b** with alkyne dipolarophiles like acetylenedicarboxylates or monocarboxylates **13a**–**d** proceeded with aromatization of the putative dihydroaromatic intermediates to produce the final cycloaddition products **14d**–**g**.

**Figure 3:**
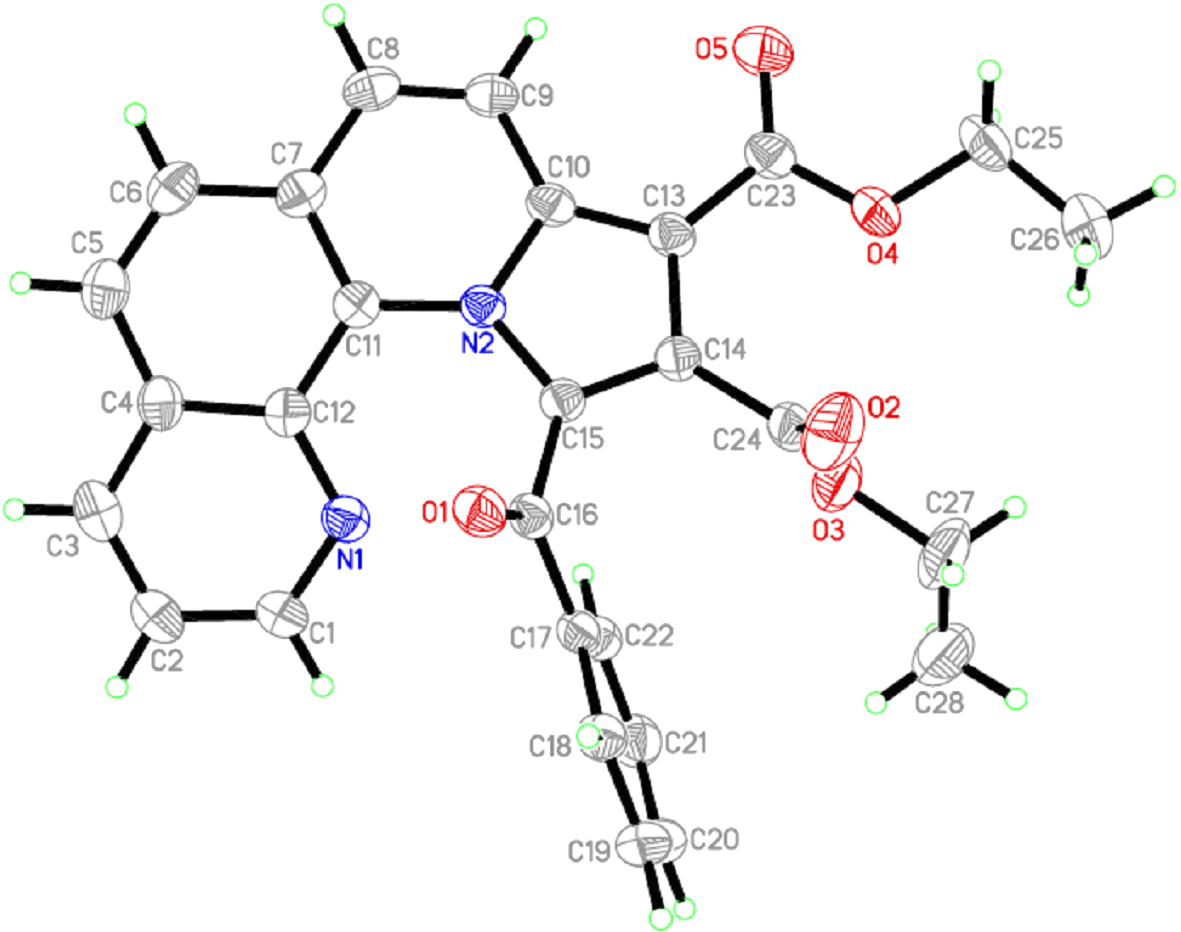
ORTEP diagram showing the molecular structure of **14e** at 30% probability level.

Characterization of the products was done via mass and NMR spectral studies. Furthermore, the single-crystal X-ray study of cycloadduct **14e** undoubtedly confirmed the structure of these cycloadducts, as obvious from the ORTEP diagram presented in Figure 3.

## Conclusion

In conclusion, a simple CuCl_2_–phenanthroline catalyzed methodology has been developed to synthesize a series of unique heteroaromatic polycycles **10a**–**h, 14a**–**g** by a 1,3-dipolar cycloaddition reaction, using furo[3,2-*h*]quinolinium/phenanthrolinium dipole precursors and maleimide/acetylenecarboxylate dipolarophiles. High atom-economy, good to very good isolated yield of the products, short reaction time and ease of separation coupled with general applicability are the key features of this methodology.

## Experimental

**General procedure to synthesize pyrrolo[3′,4′:3,4]pyrrolo[1,2-a]furoquinolines 10a–h, pyrrolo[3′,4′:3,4]pyrrolo[1,2-a]phenanthrolines 14a–c, and pyrrolo[1,2-a]phenanthrolines 14d–g:** A mixture of 3.3 mmol furo[3,2-*h*]quinolinium derivatives **9a**–**d**/phenanthroliniums **12a**,**b** and 3.3 mmol *N*-phenylmalimide derivatives **4a**–**d**/dialkyl acetylenedicarboxylates **13a**–**b**/monoalkyl acetylenemonocarboxylates **13c**,**d** was placed in a round bottomed flask (25 mL). To this MeCN (50 mL) and DBU (1 mmol) were added and the mixture was stirred for 30 min. Then 5 mol % CuCl_2_ and 5 mol % of either **L_1_**, **L_2_** were added to the reaction mixture and stirred continuously for 3 h at 65 °C. After completion of the reaction (monitored by TLC), the reaction mixture was partitioned between brine and ethyl acetate. The organic layer was then evaporated and purified by column chromatography (ethyl acetate:hexane).

**NMR data and crystal data of some representative compounds: a) spectral data of 10a:** Yellow solid. 94% yield; mp 246–248 °C; *R*_f_ (20% ethyl acetate–hexane) 0.35; ^1^H NMR (300 MHz, CDCl_3_) δ 3.65 (t, *J* = 8.1 Hz, 1H), 3.84 (s, 3H), 3.89 (m, 1H), 5.52 (m, 1H), 6.18 (m, 1H), 6.64 (s, 1H), 6.76 (s, 1H), 6.98 (m, 8H), 7.11 (m, 1H), 7.20 (m, 2H), 7.65 (t, *J* = 7.5 Hz, 2H), 7.76 (d, *J* = 7.2 Hz, 1H), 8.48 (d, *J* = 7.2 Hz, 1H); ^13^C NMR (150 MHz, CDCl_3_) δ 47. 2 (CH), 47.4 (CH), 55.5 (CH_3_), 61.0 (CH), 66.5 (CH), 101.6 (CH), 110.8 (CH), 114.5 (2CH), 116.0 (C), 119.7 (CH), 124.0 (CH), 124.6 (2CH), 127.6 (2CH), 127.6 (C), 128.4 (3CH), 128.4 (C), 128.8 (C), 129.3 (C), 129.4 (2CH), 129.6 (2CH), 131.2 (C), 133.1 (C), 134.4 (CH), 140.6 (C), 157.0 (C), 159.8 (C), 174.9 (C), 176.6 (C), 194.5 (C); HRMS (ESI) *m*/*z*: [M + Na]^+^ calcd for C_36_H_25_ClN_2_NaO_5_^+^ 623.1344; found, 623.1353. **b) Spectral data of 14a:** Yellow solid. 81% yield; mp 242–243 °C; *R*_f_ (20% ethyl acetate–hexane) 0.31; ^1^H NMR (300 MHz, CDCl_3_) δ 2.26 (s, 6H), 3.65 (m, 1H), 3.74 (m, 1H), 5.94 (m, 1H), 6.18 (m, 1H), 6.54 (m, 1H), 7.00 (m, 3H), 7.07 (m, 2H), 7.19 (m, 2H), 7.64 (m, 4H), 7.85 (m, 1H), 8.33 (m, 2H); ^13^C NMR (75 MHz, CDCl_3_) δ 19.5 (CH_3_), 19.7 (CH_3_), 46.9 (CH), 47.5 (CH), 63.4 (CH), 67.0 (CH), 116.9 (CH), 120.4 (CH), 121.1 (C), 121.6 (CH), 123.7 (CH), 126.4 (CH), 126.9 (CH), 127.3 (CH), 128.2 (C), 128.7 (2CH), 128.8 (2CH), 129.1 (C), 129.4 (C), 130.2 (CH), 132.9 (CH), 134.6 (C), 136.0 (CH), 137.7 (C), 137.9 (C), 138.5 (C), 145.3 (CH), 175.7 (C), 176.9 (C), 196.6 (C); HRMS (ESI) *m*/*z*: [M + Na]^+^ calcd for C_32_H_25_N_3_NaO_3_^+^ 522.1788; found, 522.1799. **c) Spectral data of 14e:** Brown solid. 91% yield; mp 233–234 °C; *R*_f_ (20% ethyl acetate–hexane) 0.33; ^1^H NMR (300 MHz, CDCl_3_) δ 1.06 (t, *J* = 7.2 Hz, 3H), 1.38 (t, *J* = 7.2 Hz, 3H), 3.72 (m, 1H), 3.88 (m, 1H), 4.38 (m, 2H), 7.33 (m, 1H), 7.51 (t, *J* = 7.4 Hz, 2H), 7.60 (m, 1H), 7.69 (d, *J* = 9.3 Hz, 1H), 7.83 (m, 2H), 8.01 (m, 1H), 8.17 (d, *J* = 7.8 Hz, 3H), 8.57 (d, *J* = 9.3 Hz, 1H); ^13^C NMR (75 MHz, CDCl_3_) δ 13.6 (CH_3_), 14.3 (CH_3_), 60.4 (CH_2_), 61.4 (CH_2_), 104.0 (C), 120.2 (CH), 122.5 (CH), 125.3 (CH), 125.6 (C), 126.8 (C), 126.0 (CH), 126.7 (CH), 127.7 (C), 128.1 (2CH), 129.0 (C), 130.0 (2CH), 130.7 (C), 132.2 (CH), 135.9 (CH), 137.3 (C), 137.4 (C), 137.9 (C), 145.7 (CH), 163.5 (C), 165.5 (C), 184.4 (C); HRMS (ESI) *m*/*z*: [M + Na]^+^ calcd for C_28_H_22_N_2_NO_5_^+^ 489.1421; found, 489.1437. **d) Crystal data for 10a**: C_36_H_25_N_2_O_5_Cl, *M* = 601.03 , monoclinic, *P*2_1_/*c*, *a* = 15.687(2), *b* = 19.297(2), *c* = 9.848(1) Å, β = 99.478(8)°, *V* = 2940.5(7) Å^3^, Z = 4, *D*_c_ = 1.358 g cm^−3^, µ = 0.178 mm^−1^, *F*_(000)_ = 1248, λ(MoK_α_) = 0.71073 Å, reddish block, crystal size: 0.7 × 0.25 × 0.19 mm, 37123 reflections measured (*R*_int_ = 0.0477), 3620 unique reflections, *wR(F^2^)* = 0.155 for all data and conventional *R* = 0.041 for 2932 *F*-values with *I*>2σ(*I*), (*Δ/σ*)_max_ = 0.000, S = 0.617 for all data and 398 parameters, *Δ*ρ_max, min_ (e/Å^3^) = 0.214, −0.197. **e) Crystal data for 13f:** C_28_H_22_N_2_O_5_, *M* = 466.48 , monoclinic, *P*2_1_/*c*, *a* = 11.9766(8), *b* = 17.281(1), *c* = 11.6975(8) Å, β = 109.484(3)°, *V* = 2282.4(3) Å^3^, Z = 4, *D*_c_ = 1.358 g cm^−3^, µ = 0.094 mm^−1^, *F*_(000)_ = 976, λ (Mo K_α_) = 0.71073 Å, yellowish block, crystal size: 0.17 × 0.11 × 0.09 mm, 25248 reflections measured (*R*_int_ = 0.0552), 3534 unique reflections, *wR(F^2^)* = 0.1976 for all data and conventional *R* = 0.0501 for 2741 *F*-values with *I*>2σ(*I*), (*Δ/σ*)_max_ = 0.000, S = 1.504 for all data and 319 parameters, *Δ*ρ_max, min_ (e/Å^3^) = 0.267, −0.351. Unit cell determinations and intensity data collections for both the compounds were performed on a Bruker KAPPA APEXII CCD diffractometer at 296(2) K. Structure solution by direct methods and refinements by full-matrix-least-squares methods on *F*^2^. Programs: APEX2 (Bruker AXS Inc., Madison, Wisconsin, USA) for data collection, SAINT (Bruker AXS Inc., Madison, Wisconsin, USA) for cell refinement and data reduction, SHELXTL (Bruker AXS Inc., Madison, Wisconsin, USA) for structure determination, refinements and molecular graphics calculations. CCDC Numbers: 937560 (for compound **10a**) and 937561 (for compound **14e**) contain the supplementary crystallographic data for this paper. These data can be obtained free of charge from The Cambridge Crystallographic Data Centre via http://www.ccdc.cam.ac.uk/data_request/cif (or from the Cambridge Crystallographic Data Centre, 12, Union Road, Cambridge CB2 1EZ, UK; fax: +44 1223 336033)

## Supporting Information

File 1 Experimental and analytical data and copies of ^1^H NMR and ^13^C NMR spectra of all new products.
