# Supplementary material for: Copper–phenanthroline catalysts for regioselective synthesis of pyrrolo[3′,4′:3,4]pyrrolo[1,2-a]furoquinolines/phenanthrolines and of pyrrolo[1,2-a]phenanthrolines under mild conditions
Source: Beilstein J Org Chem. 2014 Mar 20;10:692–700. doi: 10.3762/bjoc.10.62 (PMC3999842; doi:10.3762/bjoc.10.62)

Supporting Information

for

Copper–phenanthroline catalysts for regioselective synthesis of pyrrolo[3′,4′:3,4]pyrrolo[1,2-*a*]furoquinolines/ phenanthrolines and of pyrrolo[1,2-*a*]phenanthrolines under mild conditions

Rupankar Paira, Tarique Anwar, Maitreyee Banerjee, Yogesh P. Bharitkar, Shyamal Mondal, Sandip Kundu, Abhijit Hazra, Prakas R Maulik and Nirup B Mondal*

Address: Department of Chemistry, Indian Institute of Chemical Biology, Council of Scientific and Industrial Research, 4 Raja S.C. Mullick Road, Jadavpur, Kolkata-700032, India

Email: Nirup B. Mondal* - nirup@iicb.res.in

* Corresponding author

Experimental and analytical data and copies of ^{1}H NMR and ^{13}C NMR spectra of all new products

Table of contents

1. Experimental section S2–S4
2. Spectral data of all new compounds S4–S11
3. Scan copies of NMR spectra of all new compounds S11–S39

1. Experimental section:

1.1 General experimental:

Melting points were determined with a capillary melting point apparatus and are uncorrected. IR spectra were recorded on a JASCO FTIR (model 410) in KBr pellets. ESI-MS (positive) was conducted using LC-ESI-Q-TOF micro mass spectrometer (Indian Institute of Chemical Biology, Kolkata). The NMR spectra were taken on a Bruker 300/600 DPX spectrometer operating at 300/600 MHz for ^{1}H and 75/150 MHz for ^{13}C, respectively, with tetramethylsilane (TMS) as an internal standard and the chemical shifts are reported in δ units. Microwave irradiation was performed by using a mono-mode Discover microwave reactor (CEM Corp., Matthews, NC, USA). Quinoline derivatives, acetylene derivatives and ω-bromoacetophenones were purchased from Aldrich Chemical Ltd (USA). Thin-layer chromatography was performed on pre-coated silica gel 60 F254 aluminum sheets (E. Merck, Germany) using different solvent system.

1.2 General procedure for the synthesis of furo[3,2-*h*]quinoliniums 9a–d and phenanthroliniums 12a–b:

At the outset, furo[3,2-*h*]quinoline derivatives **7a–b** were synthesized according to literature procedure.[1] After that, 3.3 mmol of furo[3,2-*h*]quinoline derivatives **7a–b** or phenanthroline (**11**) and 3.3 mmol ω-bromoacetophenones **8a–c** were placed in a round bottomed flask (25 mL) and dissolved in minimum amount of DCM. Basic alumina (0.4 g) was then added to the solution and the organic solvent was evaporated to dryness under reduced pressure. After fitting the flask with a septum the mixture was subjected to irradiation in a microwave reactor (CEM, Discover, USA) at 90 °C (180 W) for appropriate amount of time (as

monitored by TLC). After completion of the reaction the reaction mixture was cooled and chloroform was added to it and the slurry was stirred at room temperature for 10 min. The mixture was then filtered through a sintered glass funnel. The filtrate was then evaporated to dryness under reduced pressure and the residue was purified by flash chromatography to isolate the product **9a–d**, **12a–b.**

1.3 General procedure for the synthesis of maleimide derivatives:

a) A three-necked flask provided with a paddle-type stirrer, a reflux condenser, and a dropping funnel are placed and 2 moles of maleic anhydride was dissolved in ethyl ether (50 mL), then 2 moles of aniline were added through the dropping funnel. The resulting thick suspension is stirred at room temperature for 1 hour and is then cooled to 15–20 °C in an ice bath. The resulting maleanilic acid is obtained by suction filtration.

b) 60 mL acetic anhydride and 4g anhydrous sodium acetate were placed in a 250 mL conical flask. The maleanilic acid obtained as described above, is added, and the resulting suspension is dissolved by swirling and heating on a steam bath for 30 minutes. The reaction mixture is cooled almost to room temperature in a cold water bath and is then poured into 1L of ice water. The precipitated product is removed by suction filtration, washed three times with ice-cold water and once with petroleum ether, and dried.

1.4 General procedure to synthesize pyrrolo[3',4':3,4]pyrrolo[1,2-*a*]furoquinolines (10a–h) and pyrrolo[3',4':3,4]pyrrolo[1,2-*a*]phenanthrolines (14a–g):

A mixture of 3.3 mmol furo[3,2-*h*]quinolinium derivatives **9a–d** / phenanthroliniums **12a–b** and 3.6 mmol *N*-phenylmaleimide derivatives (**4a–d**) / dialkylacetylene diacetates (**13a–**

b) / monoalkyl acetylene monoacetates (**13c–d**) were placed in a round bottomed flask (25 mL). Then MeCN (50 mL) and DBU (1 mmol) were added and the mixture was stirred for 30 min. Then 5 mol % $CuCl_2$ and 5 mol % of either $\mathbf{L_1}$, $\mathbf{L_2}$ and 3.6 mmol *N*-phenyl maleimide derivatives (**4a–d**) / dialkyl acetylene diacetates (**13a–b**) / monoalkyl acetylene monoacetates (**13c–d**) were then added to the reaction mixture and stirred continuously for 3 h at 65 °C. After completion of the reaction (monitored by TLC), the reaction mixture was extracted with brine and ethyl acetate. The organic layer was evaporated and purified by column chromatography (ethyl acetate:hexane).

2. Spectral data of all new compounds:

2.1 Spectral data of 10a: Yellow solid. 94% yield; Mp 246–248°C; R_f (20% ethyl acetate–hexane) 0.35; ^{1}HNMR (300 MHz, $CDCl_3$): δ 3.65 (t, *J* = 8.1 Hz., 1H), 3.84 (s, 3H), 3.89 (m, 1H), 5.52 (m, 1H), 6.18 (m, 1H), 6.64 (s, 1H), 6.76 (s, 1H), 6.98 (m, 8H), 7.11 (m, 1H), 7.20 (m, 2H), 7.65 (t, *J* = 7.5 Hz., 2H), 7.76 (d, *J* = 7.2 Hz., 1H), 8.48 (d, *J* = 7.2 Hz., 2H); ^{13}CNMR (150 MHz., $CDCl_3$): δ 47. 2 (CH), 47.4 (CH), 55.5 (CH_3), 61.0 (CH), 66.5 (CH), 101.6 (CH), 110.8 (CH), 114.5 (2CH), 116.0 (C), 119.7 (CH), 124.0 (CH), 124.6 (2CH), 127.6 (2CH), 127.6 (C), 128.4 (3CH), 128.4 (C), 128.8 (C), 129.3 (C), 129.4 (2CH), 129.6 (2CH), 131.2 (C), 133.1 (C), 134.4 (CH), 140.6 (C), 157.0 (C), 159.8 (C), 174.9 (C), 176.6 (C), 194.5 (C); HRMS (ESI) m/z calcd for $C_{36}H_{25}ClN_2NaO_5^+$: $[M+Na]^+$ 623.1344; found: 623.1353.

2.2 Spectral data of 10b: Brown solid. 84% yield; Mp 248–250°C; R_f (20% ethyl acetate–hexane) 0.35; ^{1}HNMR (600 MHz, $CDCl_3$): δ 2.24 (s, 3H), 3.64 (m, 1H), 3.85 (m, 4H), 5.50 (d, *J* = 7.8 Hz., 1H), 6.16 (m, 1H), 6.64 (s, 1H), 6.70 (s, 1H), 6.74 (m, 2H), 6.95 (m, 5H), 7.20 (d, *J* = 9.0 Hz., 2H), 7.44 (m, 1H), 7.66 (t, *J* = 7.2 Hz., 2H), 7.78 (t, *J* = 7.2 Hz., 1H), 8.47 (d, *J* = 7.2

Hz., 2H); ^{13}CNMR (150 MHz., $CDCl_3$): δ 21.5 (CH_3), 47.2 (CH), 47.4 (CH), 55.5 (CH_3), 61.0 (CH), 66.5 (CH), 101.2 (CH), 110.7 (CH), 114.5 (2CH), 115.7 (C), 119.5 (CH), 124.0 (CH), 124.5 (2CH), 125.2 (CH), 126.5 (C), 127.5 (2CH), 127.6 (C), 128.1 (CH), 128.7 (C), 129.1 (C), 129.4 (2CH), 129.6 (2CH), 131.3 (C), 133.1 (C), 134.3 (CH), 138.6 (C), 140.5 (C), 157.3 (C), 159.7 (C), 174.9 (C), 176.6 (C), 194.5 (C). HRMS (ESI) m/z calcd for $C_{37}H_{27}ClN_2NaO_5^+$: $[M+Na]^+$ 637.1501; found: 637.1510.

2.3 Spectral data of 10c: Yellow solid. 91% yield; Mp 246–247°C; R_f (20% ethyl acetate–hexane) 0.33; ^{1}HNMR (300 MHz, $CDCl_3$): δ 2.29 (s, 6H), 3.63 (d, *J* = 8.1 Hz., 1H), 3.88 (m, 1H), 5.50 (d, *J* = 8.4 Hz., 1H), 6.17 (m, 1H), 6.64 (s, 1H), 6.75 (s, 1H), 6.96 (m, 7H), 7.18 (m, 3H), 7.61 (m, 2H), 7.74 (m, 1H), 8.47 (d, *J* = 7.2 Hz., 2H); ^{13}CNMR (75 MHz., $CDCl_3$): δ 19.5 (CH_3), 19.8 (CH_3), 47.2 (CH), 47.4 (CH), 61.0 (CH), 66.6 (CH), 101.5 (CH), 110.8 (CH), 116.0 (C), 119.7 (CH), 123.7 (CH), 124.0 (CH), 124.5 (2CH), 127.2 (CH), 127.3 (C), 127.6 (CH), 127.8 (C), 128.4 (2CH), 128.7 (C), 128.9 (C), 129.3 (2CH), 129.4 (2CH), 129.6 (C), 130.3 (CH), 131.2 (C), 133.2 (C), 134.3 (CH), 137.9 (C), 140.6 (C), 157.0 (C), 174.8 (C), 176.6 (C), 194.5 (C). HRMS (ESI) m/z calcd for $C_{37}H_{27}ClN_2NaO_4^+$: $[M+Na]^+$ 621.1552; found: 621.1567.

2.4 Spectral data of 10d: Yellow solid. 89% yield; Mp 237–238°C; R_f (20% ethyl acetate–hexane) 0.36; ^{1}HNMR (300 MHz, $CDCl_3$): δ 2.29 (s, 6H), 2.40 (s, 3H), 3.76 (m, 1H), 3.98 (m, 1H), 4.50 (d, *J* = 6.0 Hz., 1H), 4.41 (m, 1H), 7.17 (s, 1H), 7.25 (m, 5H), 7.43 (m, 1H), 7.52 (m, 2H), 7.79 (d, *J* = 8.7 Hz., 1H), 7.85 (s, 1H), 7.90 (d, *J* = 8.1 Hz., 2H), 8.02 (d, *J* = 7.5 Hz., 2H), 8.67 (d, *J* = 8.4 Hz., 1H); ^{13}CNMR (75 MHz., $CDCl_3$): δ 14.1 (CH_3), 19.5 (CH_3), 19.8 (CH_3), 37.9 (CH), 40.7 (CH), 50.7 (CH), 53.7 (CH), 101.8 (CH), 120.3 (CH), 122.0 (CH), 123.2 (C), 124.3 (CH), 125.2 (2CH), 126.1 (C), 127.8 (CH), 128.3 (2CH), 128.9 (2CH), 129.1 (C), 129.3 (CH), 129.4 (2CH), 129.8 (C), 129.9 (CH), 133.6 (C), 134.8 (CH), 136.2 (C), 137.5 (C), 137.7

(C), 144.7 (C), 148.0 (C), 155.9 (C), 157.7 (C), 175.3 (C), 178.6 (C), 197.4 (C). HRMS (ESI) m/z calcd for $C_{38}H_{29}ClN_2NaO_4^+$: $[M+Na]^+$ 635.1708; found: 635.1723.

2.5 Spectral data of 10e: Yellow solid. 89% yield; Mp 233–235°C; R_f (20% ethyl acetate–hexane) 0.33; ^{1}HNMR (300 MHz, $CDCl_3$): δ 2.65 (s, 6H), 3.64 (m, 1H), 3.82 (m, 1H), 5.48 (d, *J* = 8.1 Hz., 1H), 6.18 (m, 1H), 6.56 (s, 1H), 6.75 (s, 1H), 6.99 (m, 6H), 7.17 (m, 4H), 7.60 (d, *J* = 8.4 Hz., 2H), 8.40 (d, *J* = 8.4 Hz., 2H); ^{13}CNMR (150 MHz., $CDCl_3$): δ 19.6 (CH_3), 19.8 (CH_3), 47.1 (CH), 47.2 (CH), 61.1 (CH), 66.2 (CH), 101.7 (CH), 111.0 (CH), 120.4 (C), 123.7 (C), 124.3 (CH), 124.5 (CH), 125.2 (CH), 127.8 (CH), 128.3 (CH), 128.7 (C), 128.8 (C), 128.9 (CH), 129.0 (2CH), 129.1 (2CH), 129.6 (C), 129.8 (C), 129.9 (C), 130.3 (C), 130.4 (CH), 130.9 (CH), 131.6 (CH), 134.9 (CH), 137.5 (C), 140.3 (C), 155.3 (C), 157.8 (C), 175.1 (C), 178.3 (C), 196.6 (C); HRMS (ESI) m/z calcd for $C_{37}H_{26}Cl_2N_2NaO_4^+$: $[M+Na]^+$ 655.1162; found: 655.1174.

2.6 Spectral data of 10f: Brown solid. 85% yield; Mp 234–236°C; R_f (20% ethyl acetate–hexane) 0.34; ^{1}HNMR (300 MHz, $CDCl_3$): δ 2.26 (s, 3H), 2.30 (s, 3H), 3.80 (m, 1H), 3.98 (m, 1H), 4.41 (d, *J* = 8.1 Hz., 1H), 4.65 (m, 1H), 7.16 (m, 3H), 7.29 (s, 1H), 7.50 (m, 7H), 7.85 (s, 1H), 7.98 (m, 4H), 8.67 (d, *J* = 8.4 Hz., 1H); HRMS (ESI) m/z calcd for $C_{37}H_{27}ClN_2NaO_4^+$: $[M+Na]^+$ 621.1552; found: 621.1558.

2.7 Spectral data of 10g: Yellow solid. 87% yield; Mp 241–243°C; R_f (20% ethyl acetate–hexane) 0.35; ^{1}HNMR (300 MHz, $CDCl_3$): δ 2.26 (s, 3H), 2.30 (s, 3H), 2.39 (s, 3H), 3.80 (m, 1H), 3.98 (m, 1H), 4.40 (m, 1H), 4.64 (m, 1H), 7.11 (s, 1H), 7.29 (m, 5H), 7.50 (m, 2H), 7.60 (m, 1H), 7.68 (m, 1H), 7.77 (m, 1H), 7.88 (m, 3H), 8.03 (d, *J* = 7.5 Hz., 2H), 8.66 (d, *J* = 8.4 Hz., 1H); HRMS (ESI) m/z calcd for $C_{38}H_{29}ClN_2NaO_4^+$: $[M+Na]^+$ 635.1708; found: 635.1719.

2.8 Spectral data of 10h: Yellow solid. 89% yield; Mp 244–246°C; R_f (20% ethyl acetate–hexane) 0.36; ^{1}HNMR (300 MHz, $CDCl_3$): δ 2.26 (s, 3H), 2.30 (s, 3H), 2.42 (s, 3H), 3.77 (m, 1H), 3.94 (m, 1H), 4.39 (m, 1H), 4.64 (m, 1H), 7.16 (m, 3H), 7.47 (m, 4H), 7.69 (d, *J* = 8.4 Hz., 1H), 7.82 (m, 7H), 8.66 (d, *J* = 8.4 Hz., 1H); ^{13}CNMR (150 MHz., $CDCl_3$): δ 14.3 (CH_3), 20.4 (CH_3), 21.7 (CH_3), 41.4 (CH), 42.8 (CH), 53.4 (CH), 54.5 (CH), 101.8 (CH), 120.3 (CH), 121.8 (CH). 122.1 (CH), 123.2 (C), 125.3 (2CH), 125.8 (CH), 126.2 (C), 126.3 (CH), 128.3 (2CH), 128.9 (2CH), 129.1 (C), 129.4 (CH), 129.9 (C), 131.0 (CH), 131.5 (C), 133.6 (C), 134.8 (CH), 135.0 (CH), 136.7 (C), 138.2 (C), 138.5 (C), 144.7 (C), 148.1 (C), 157.7 (C), 175.3 (C), 178.2 (C), 197.0 (C). HRMS (ESI) m/z calcd for $C_{38}H_{29}ClN_2NaO_4^+$: $[M+Na]^+$ 635.1714; found: 635.1729.

2.9 Spectral data of 14a : Yellow solid. 81% yield; Mp 242–243°C; R_f (20% ethyl acetate–hexane) 0.31; ^{1}HNMR (300 MHz, $CDCl_3$): δ 2.26 (s, 6H), 3.65 (m, 1H), 3.74 (m, 1H), 5.94 (m, 1H), 6.18 (m, 1H), 6.54 (m, 1H), 7.00 (m, 3H), 7.07 (m, 2H), 7.19 (m, 2H), 7.64 (m, 4H), 7.85 (m, 1H), 8.33 (m, 2H); ^{13}CNMR (75 MHz., $CDCl_3$): δ 19.5 (CH_3), 19.7 (CH_3), 46.9 (CH), 47.5 (CH), 63.4 (CH), 67.0 (CH), 116.9 (CH), 120.4 (CH), 121.1 (C), 121.6 (CH), 123.7 (CH), 126.4 (CH), 126.9 (CH), 127.3 (CH), 128.2 (C), 128.7 (2CH), 128.8 (2CH), 129.1 (C), 129.4 (C), 130.2 (CH), 132.9 (CH), 134.6 (C), 136.0 (CH), 137.7 (C), 137.9 (C), 138.5 (C), 145.3 (CH), 175.7 (C), 176.9 (C), 196.6 (C); HRMS (ESI) m/z calcd for $C_{32}H_{25}N_3NaO_3^+$: $[M+Na]^+$ 522.1788 found: 522.1799.

2.10 Spectral data of 14b : Yellow solid. 83% yield; Mp 240–243°C; R_f (20% ethyl acetate–hexane) 0.32; ^{1}HNMR (300 MHz, $CDCl_3$): δ 3.65 (m, 1H), 3.74 (m, 1H), 3.81 (m, 3H), 5.94 (m, 1H), 6.18 (m, 1H), 6.54 (m, 1H), 6.95 (m, 3H), 7.07 (m, 2H), 7.19 (m, 3H), 7.62 (m, 4H), 7.86 (m, 1H), 8.33 (m, 2H); ^{13}CNMR (75 MHz., $CDCl_3$): δ 46.8 (CH), 47.5 (CH), 55.4 (CH_3), 63.5

(CH), 67.0 (CH), 114.4 (2CH), 117.0 (CH), 120.4 (CH), 121.1 (C), 121.6 (CH), 124.2 (C), 126.4 (CH), 127.0 (CH), 127.6 (2CH), 128.8 (2CH), 128.9 (2CH), 129.5 (C), 133.0 (CH), 134.6 (C), 136.0 (CH), 137.9 (C), 138.5 (C), 145.4 (CH), 159.6 (C), 175.7 (C), 177.0 (C), 196.6 (C). HRMS (ESI) m/z calcd for $C_{31}H_{23}N_3NaO_4^+$: $[M+Na]^+$ 524.1581 found: 524.1596

2.11 Spectral data of 14c : Brown solid. 88% yield; Mp 248–250°C; R_f (20% ethyl acetate–hexane) 0.32; ^{1}HNMR (300 MHz, $CDCl_3$): δ 3.65 (t, *J* = 8.1 Hz., 1H), 3.82 (m, 4H), 5.46 (m, 1H), 6.20 (m, 1H), 6.52 (s, 1H), 6.76 (s, 1H), 6.98 (m, 3H), 7.06 (m, 3H), 7.18 (m, 3H), 7.70 (d, *J* = 8.4 Hz., 1H), 8.30 (m, 1H), 8.53 (s, 1H); ^{13}CNMR (75 MHz., $CDCl_3$): δ 47.0 (2CH), 55.5 (CH_3), 61.1 (CH), 66.4 (CH), 101.8 (CH), 111.2 (CH), 114.5 (2CH), 116.1 (C), 119.7 (CH), 123.8 (C), 124.0 (CH), 124.5 (2CH), 127.6 (2CH), 128.4 (2CH), 128.8 (CH), 129.2 (C), 131.3 (CH), 132.9 (C), 134.3 (C), 139.1 (C), 140.5 (C), 157.1 (C), 159.8 (C), 174.6 (C), 176.5 (C), 192.7 (C). HRMS (ESI) m/z calcd for $C_{31}H_{21}Cl_2N_3NaO_4^+$: $[M+Na]^+$ 592.0801; found: 592.0808.

2.12 Spectral data of 14d : Brown solid. 89% yield; Mp 231–233°C; R_f (20% ethyl acetate–hexane) 0.34; ^{1}HNMR (600 MHz, $CDCl_3$): δ 3.69 (s, 3H), 3.78 (s, 3H), 7.36 (m, 1H), 7.45 (t, *J* = 8.4 Hz., 2H), 7.51 (t, *J* = 7.8 Hz., 1H), 7.57 (m, 2H), 7.67 (m, 2H), 7.90 (m, 1H), 8.14 (t, *J* = 8.7 Hz., 2H), 8.57 (d, *J* = 9.6 Hz., 1H); ^{13}CNMR (150 MHz., $CDCl_3$): δ 52.1 (CH_3), 52.6 (CH_3), 103.9 (C), 120.8 (CH), 121.8 (CH), 125.4 (CH), 126.1 (2C), 126.7 (CH), 126.8 (CH), 128.1 (C), 128.8 (2CH), 129.3 (C), 129.8 (2CH), 130.3 (C), 133.2 (CH), 135.8 (C), 136.0 (C), 136.3 (CH), 137.5 (C), 146.4 (CH), 163.9 (C), 165.9 (C), 184.6 (C). HRMS (ESI) m/z calcd for $C_{26}H_{18}N_2NaO_4^+$: $[M+Na]^+$ 461.1108; found: 461.1103.

2.13 Spectral data of 14e : Brown solid. 91% yield; Mp 233–234°C; R_f (20% ethyl acetate–hexane) 0.33; ^{1}HNMR (300 MHz, $CDCl_3$): δ 1.06 (t, *J* = 7.2 Hz., 3H), 1.38 (t, *J* = 7.2 Hz., 3H),

3.72 (m, 1H), 3.88 (m, 1H), 4.38 (m, 2H), 7.33 (m, 1H), 7.51 (t, J = 7.4 Hz., 2H), 7.60 (m, 1H), 7.69 (d, J = 9.3 Hz., 1H), 7.83 (m, 2H), 8.01 (m, 1H), 8.17 (d, J = 7.8 Hz., 3H), 8.57 (d, J = 9.3 Hz., 1H); ^{13}CNMR (75 MHz., $CDCl_3$): δ 13.6 (CH_3), 14.3 (CH_3), 60.4 (CH_2), 61.4 (CH_2), 104.0 (C), 120.2 (CH), 122.5 (CH), 125.3 (CH), 125.6 (C), 126.8 (C), 126.0 (CH), 126.7 (CH), 127.7 (C), 128.1 (2CH), 129.0 (C), 130.0 (2CH), 130.7 (C), 132.2 (CH), 135.9 (CH), 137.3 (C), 137.4 (C), 137.9 (C), 145.7 (CH), 163.5 (C), 165.5 (C), 184.4 (C). HRMS (ESI) m/z calcd for $C_{28}H_{22}N_2NO_5^+$: $[M+Na]^+$ 489.1421; found: 489.1437.

2.14 Spectral data of 14f : Brown solid. 92% yield; Mp 236–238°C; R_f (20% ethyl acetate–hexane) 0.32; ^{1}HNMR (300 MHz, $CDCl_3$): δ 1.42 (t, J = 7.2 Hz., 3H), 4.41 (m, 2H), 7.36 (m, 1H), 7.57 (m, 3H), 7.65 (d, J = 7.2 Hz., 1H), 7.72 (d, J = 9.3 Hz., 1H), 7.81 (d, J = 8.7 Hz., 1H), 7.89 (d, J = 8.7 Hz., 1H), 8.22 (m, 4H), 8.59 (d, J = 9.3 Hz., 1H); ^{13}CNMR (75 MHz., $CDCl_3$): δ 14.5 (CH_3), 60.0 (CH_2), 106.1 (C), 119.9 (CH), 121.7 (CH), 122.4 (CH), 124.9 (CH), 125.4 (C), 125.7 (CH), 126.5 (CH), 127.6 (C), 128.3 (2CH), 129.5 (C), 130.2 (2CH), 132.4 (CH), 132.7 (C), 135.7 (CH), 137.5 (C), 138.0 (C), 138.7 (C), 146.1 (CH), 164.6 (C), 185.2 (C); HRMS (ESI) m/z calcd for $C_{25}H_{18}N_2NaO_3^+$: $[M+Na]^+$ 417.1210; found: 417.1223.

2.15 Spectral data of 14g : Yellow solid. 90% yield; Mp 240–242°C; R_f (20% ethyl acetate–hexane) 0.33; ^{1}HNMR (300 MHz, $CDCl_3$): δ 3.93 (s, 3H), 7.36 (m, 1H), 7.57 (m, 3H), 7.65 (d, J = 7.2 Hz., 1H), 7.73 (d, J = 9.3 Hz., 1H), 7.81 (d, J = 8.7 Hz., 1H), 7.89 (d, J = 8.4 Hz., 1H), 8.22 (m, 4H), 8.58 (d, J = 9.3 Hz., 1H); ^{13}CNMR (75 MHz., $CDCl_3$): δ 51.3 (CH_3), 105.7 (C), 119.8 (CH), 121.7 (CH), 122.4 (CH), 125.0 (CH), 125.4 (C), 125.8 (CH), 126.5 (CH), 127.6 (C), 128.3 (2CH), 129.4 (C), 130.2 (2CH), 132.4 (CH), 132.7 (C), 135.7 (CH), 137.4 (C), 138.0 (C), 138.7 (C), 146.1 (CH), 165.0 (C), 185.2 (C); HRMS (ESI) m/z calcd for $C_{24}H_{16}N_2NaO_3^+$: $[M+Na]^+$ 403.1053; found: 403.1058.

2.16 Crystal data for 10a: $C_{36}H_{25}N_2O_5Cl$, *M*=601.03 , monoclinic, *P*2$_1$/*c*, *a*=15.687(2), *b*=19.297(2), *c*=9.848(1)Å, β=99.478(8)°,*V*=2940.5(7)Å^3, Z=4, D_c=1.358 g cm^{-3}, μ=0.178mm^{-1}, $F_{(000)}$=1248, λ(MoK_α) = 0.71073 Å, reddish block, crystal size: 0.7 x 0.25 x 0.19 mm, 37123 reflections measured (R_{int}=0.0477), 3620 unique reflections, $wR(F^2)$ = 0.155 for all data and conventional *R* = 0.041 for 2932 *F*-values with $I>2\sigma(I)$, $(\Delta/\sigma)_{max}$=0.000, S=0.617 for all data and 398 parameters, $\Delta\rho_{max,\ min}$ (e/Å^3) = 0.214, -0.197. Crystal data for **13f**: $C_{28}H_{22}N_2O_5$, *M*=466.48 , monoclinic, *P*2$_1$/*c*, *a*=11.9766(8), *b*=17.281(1), *c*=11.6975(8)Å, β=109.484(3)°,*V*=2282.4(3)Å^3, Z=4, D_c=1.358 g cm^{-3}, μ=0.094mm^{-1}, $F_{(000)}$=976, λ(MoK_α) = 0.71073 Å, yellowish block, crystal size: 0.17 x 0.11 x 0.09 mm, 25248 reflections measured (R_{int}=0.0552), 3534 unique reflections, $wR(F^2)$ = 0.1976 for all data and conventional *R* = 0.0501 for 2741 *F*-values with $I>2\sigma(I)$, $(\Delta/\sigma)_{max}$=0.000, S=1.504 for all data and 319 parameters, $\Delta\rho_{max,\ min}$ (e/Å^3) = 0.267, -0.351.Unit cell determinations and intensity data collections for both the compounds were performed on a Bruker KAPPA APEXII CCD diffractometer at 296(2) K. Structure solution by direct methods and refinements by full-matrix-least-squares methods on F^2. Programs: APEX2 (Bruker AXS Inc., Madision, Wisconsin, USA) for data collection, SAINT (Bruker AXS Inc., Madision, Wisconsin, USA) for cell refinement and data reduction, SHELXTL (Bruker AXS Inc., Madision, Wisconsin, USA) for structure determination, refinements and molecular graphics calculations. CCDC Numbers: 937560 (for compound **10a**) and 937561 (for compound **14e**) contain the supplementary crystallographic data for this paper. These data can be obtained free of charge from The Cambridge Crystallographic Data Centre via www.ccdc.cam.ac.uk/data_request/cif.

2.17 References

1. Saha, P.; Naskar, S.; Paira, R.; Mondal, S.; Maity, A.; Sahu, K. B.; Paira, P.; Hazra, A.; Bhattacharya, D.; Banerjee, S.; Mondal, N. B. *Synthesis*, **2010**, 486-492;

3. Copies of NMR spectra of all new compounds:

^{1}H NMR of **10a**:

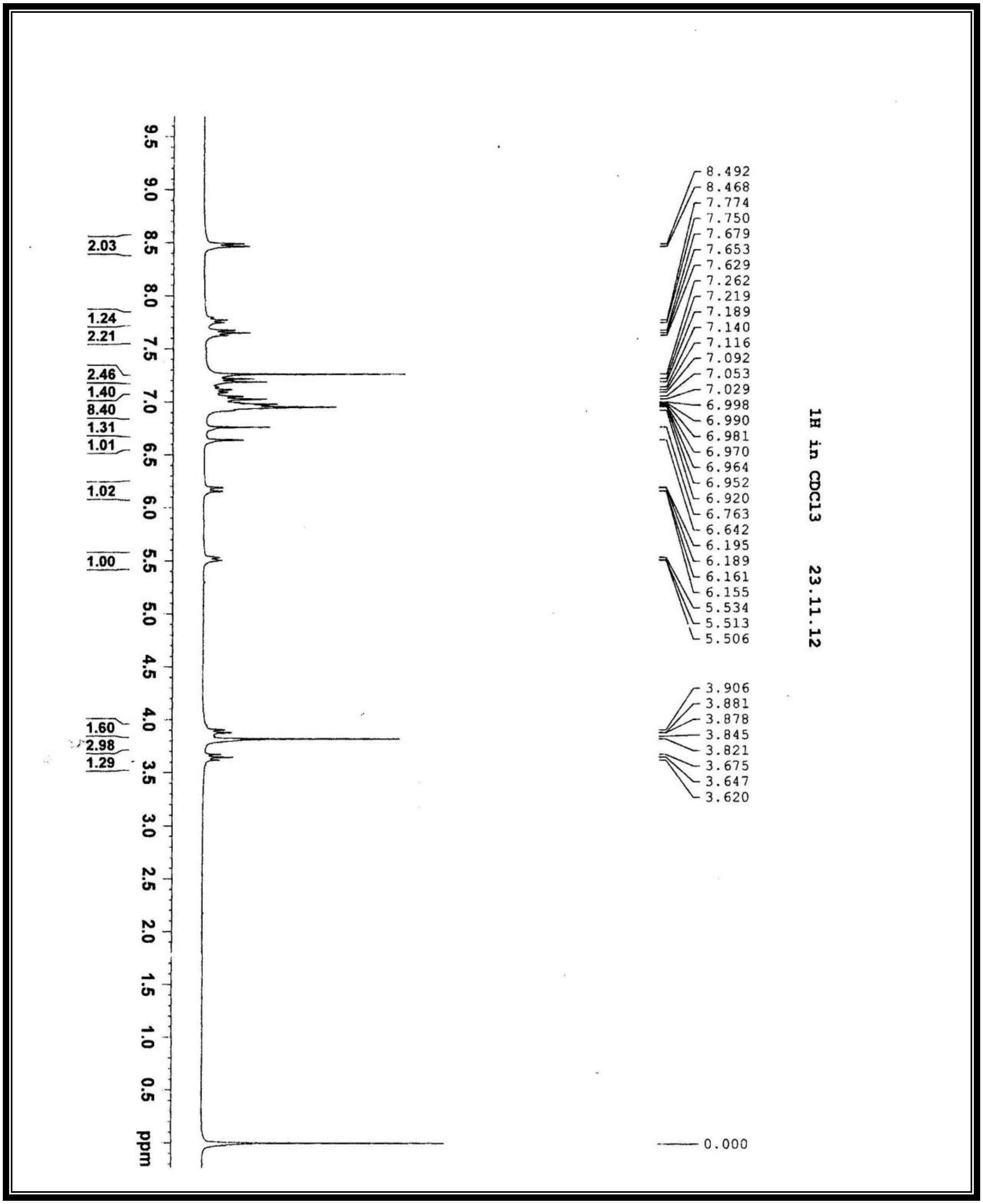

^{13}C NMR of **10a**:

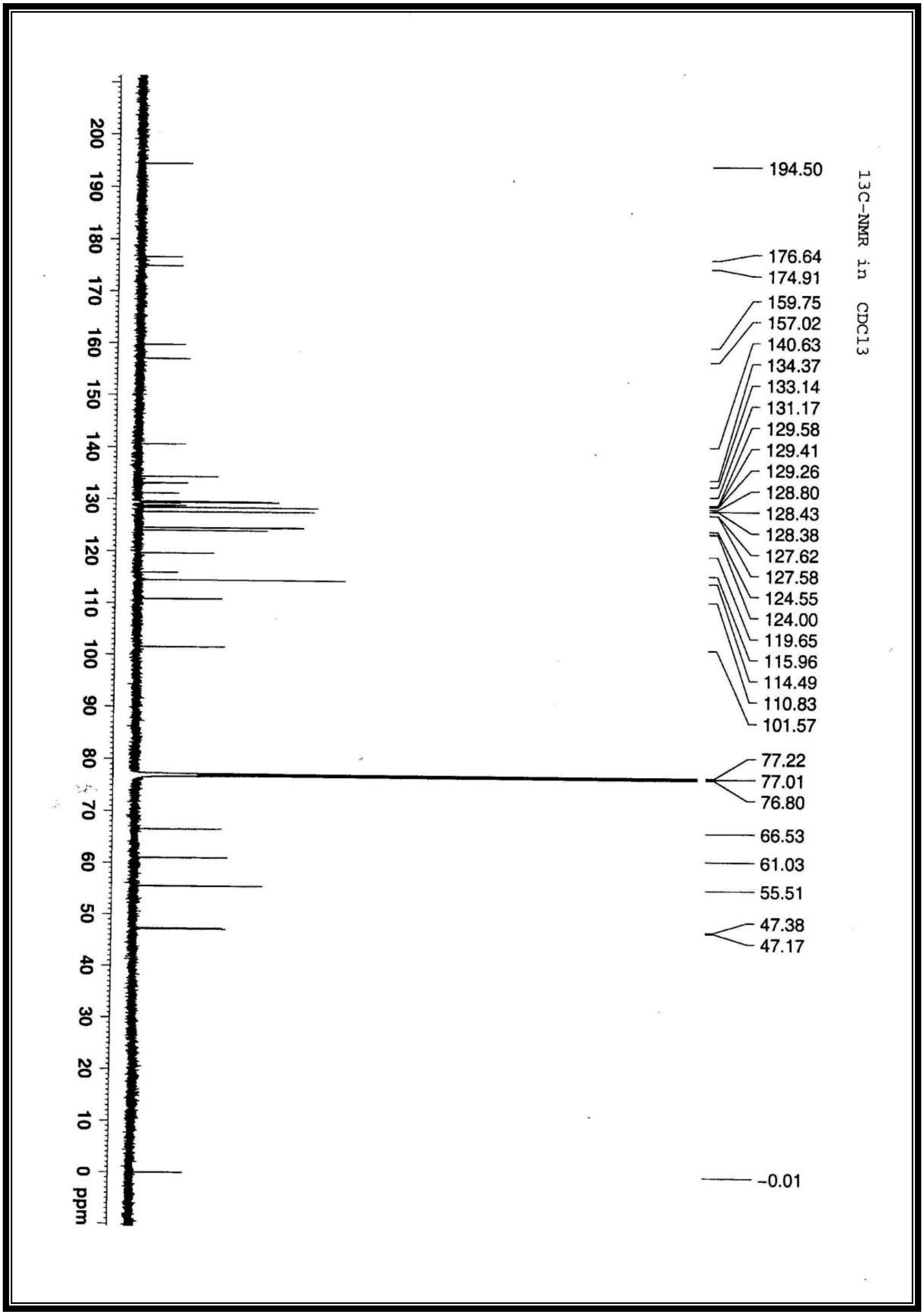

^{1}H NMR of **10b**:

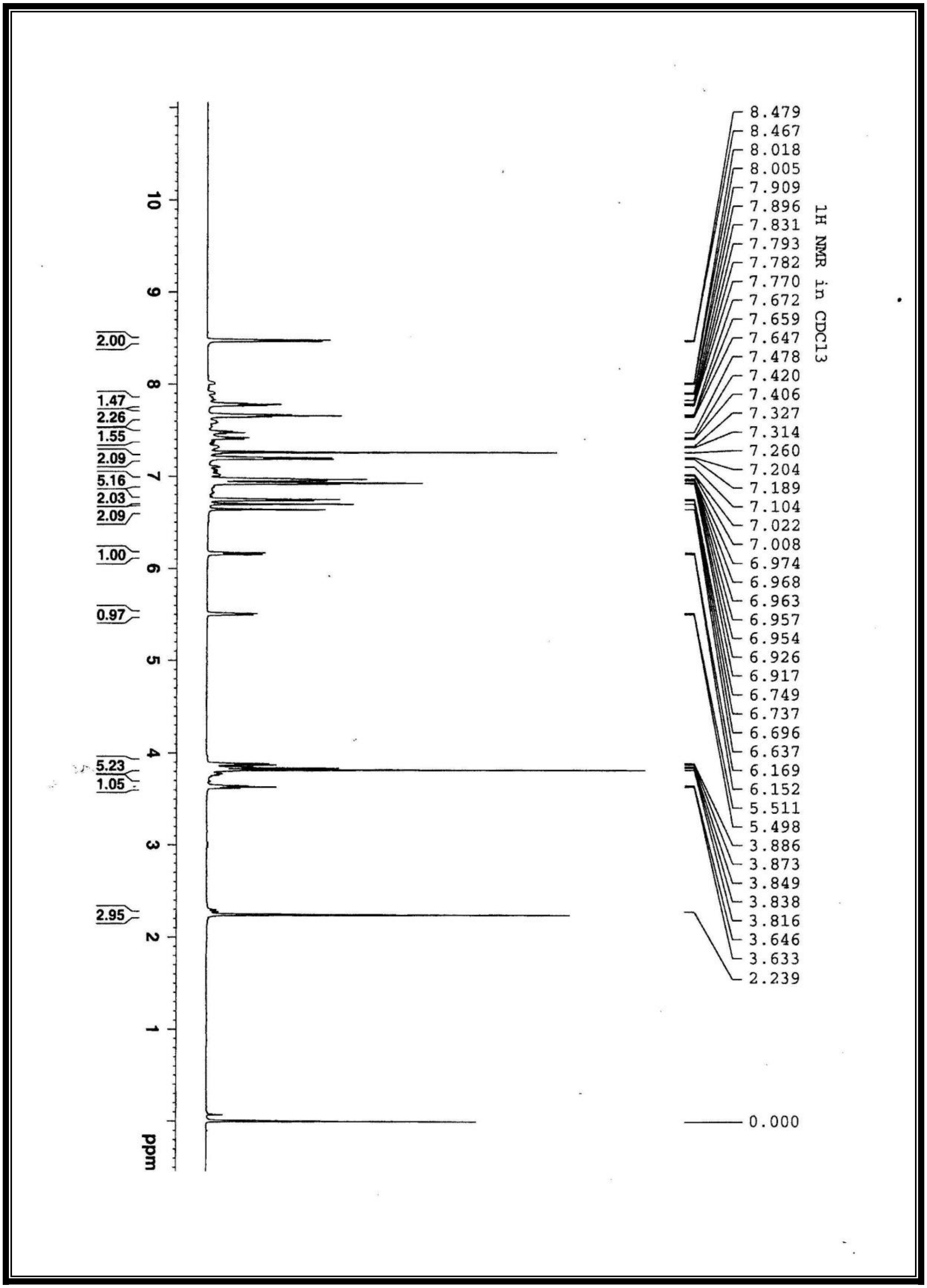
1H NMR in CDCl3
8.479
8.467
8.018
8.005
7.909
7.896
7.831
7.793
7.782
7.770
7.672
7.659
7.647
7.478
7.420
7.406
7.327
7.314
7.260
7.204
7.189
7.104
7.022
7.008
6.974
6.968
6.963
6.957
6.954
6.926
6.917
6.749
6.737
6.696
6.637
6.169
6.152
5.511
5.498
3.886
3.873
3.849
3.838
3.816
3.646
3.633
2.239
0.000
2.00
1.47
2.26
1.55
2.09
5.16
2.03
2.09
1.00
0.97
5.23
1.05
2.95
ppm

^{13}C NMR of **10b**:

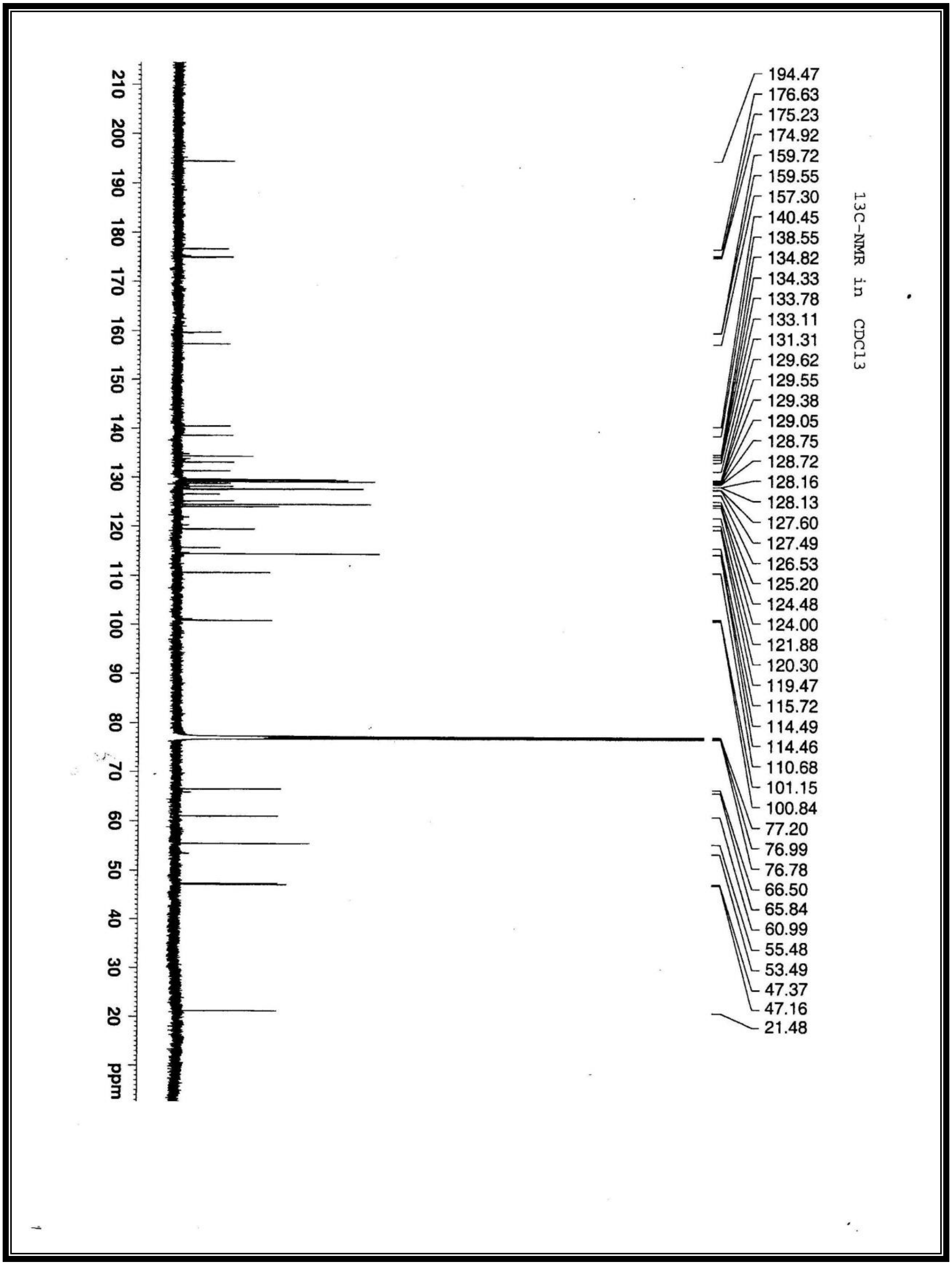

^{1}H NMR of **10c**:

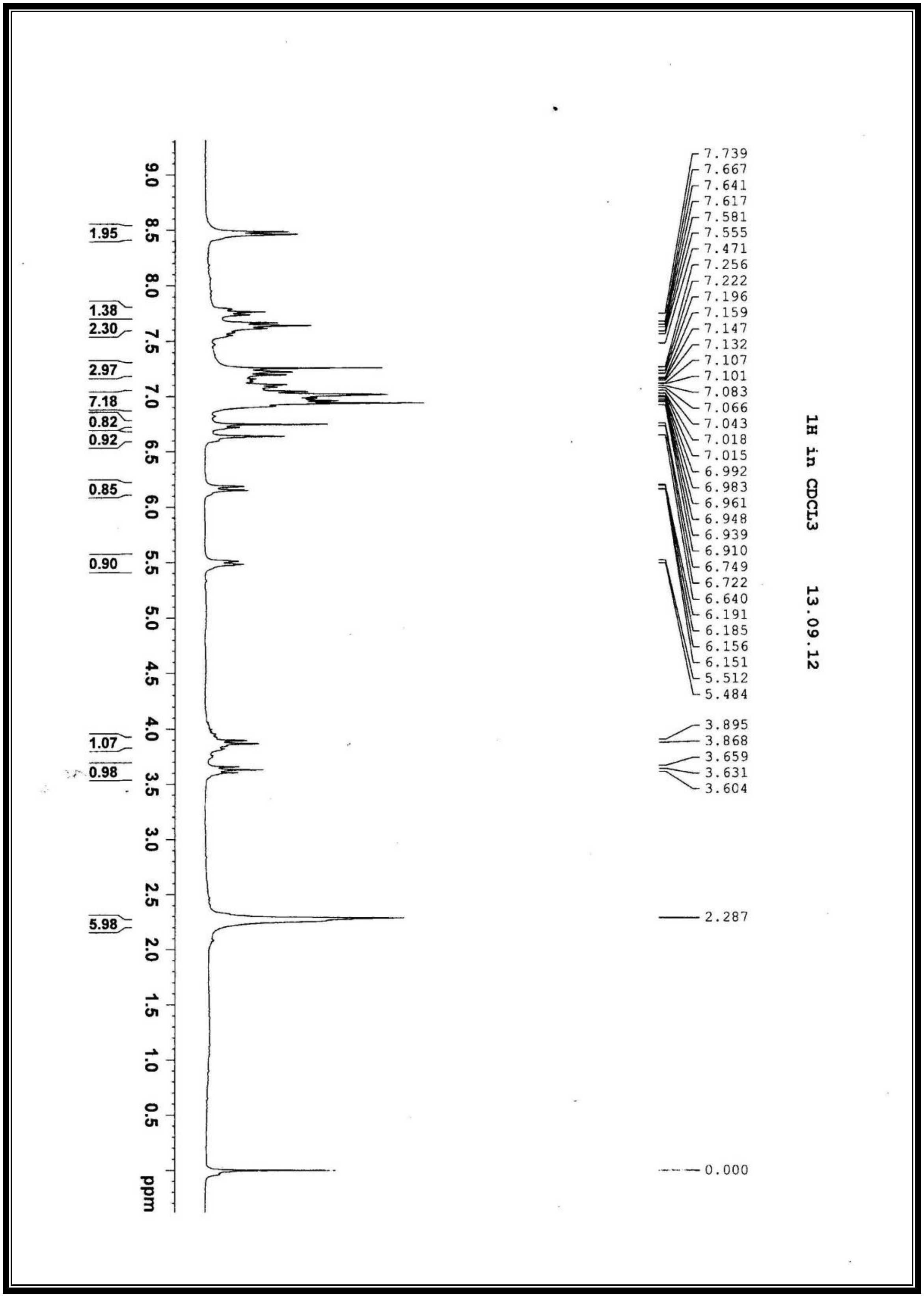
1H in CDCL3 13.09.12
7.739
7.667
7.641
7.617
7.581
7.555
7.471
7.256
7.222
7.196
7.159
7.147
7.132
7.107
7.101
7.083
7.066
7.043
7.018
7.015
6.992
6.983
6.961
6.948
6.939
6.910
6.749
6.722
6.640
6.191
6.185
6.156
6.151
5.512
5.484
3.895
3.868
3.659
3.631
3.604
2.287
0.000
1.95
1.38
2.30
2.97
7.18
0.82
0.92
0.85
0.90
1.07
0.98
5.98
9.0
8.5
8.0
7.5
7.0
6.5
6.0
5.5
5.0
4.5
4.0
3.5
3.0
2.5
2.0
1.5
1.0
0.5
ppm

^{13}C NMR of **10c**:

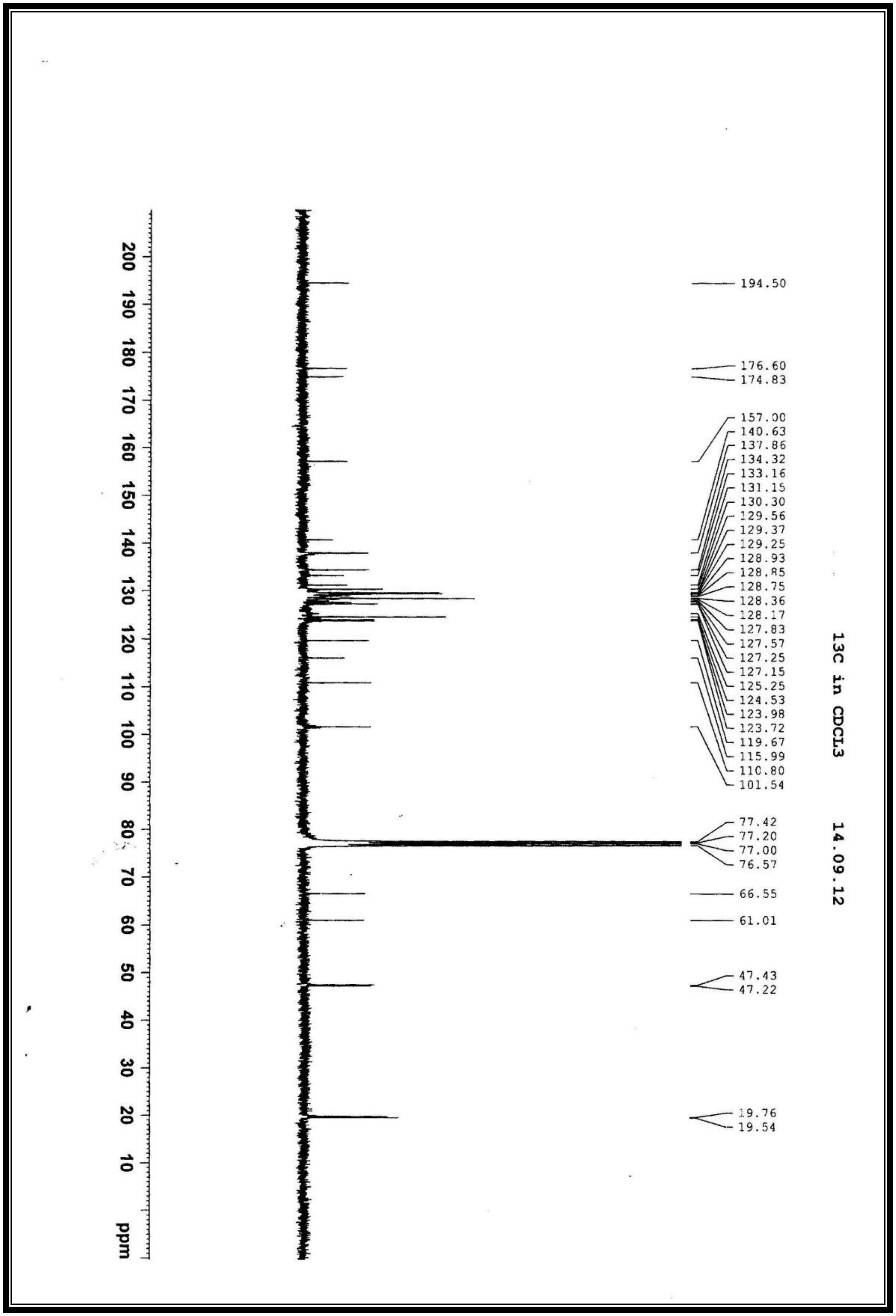

^{1}H NMR of **10d**:

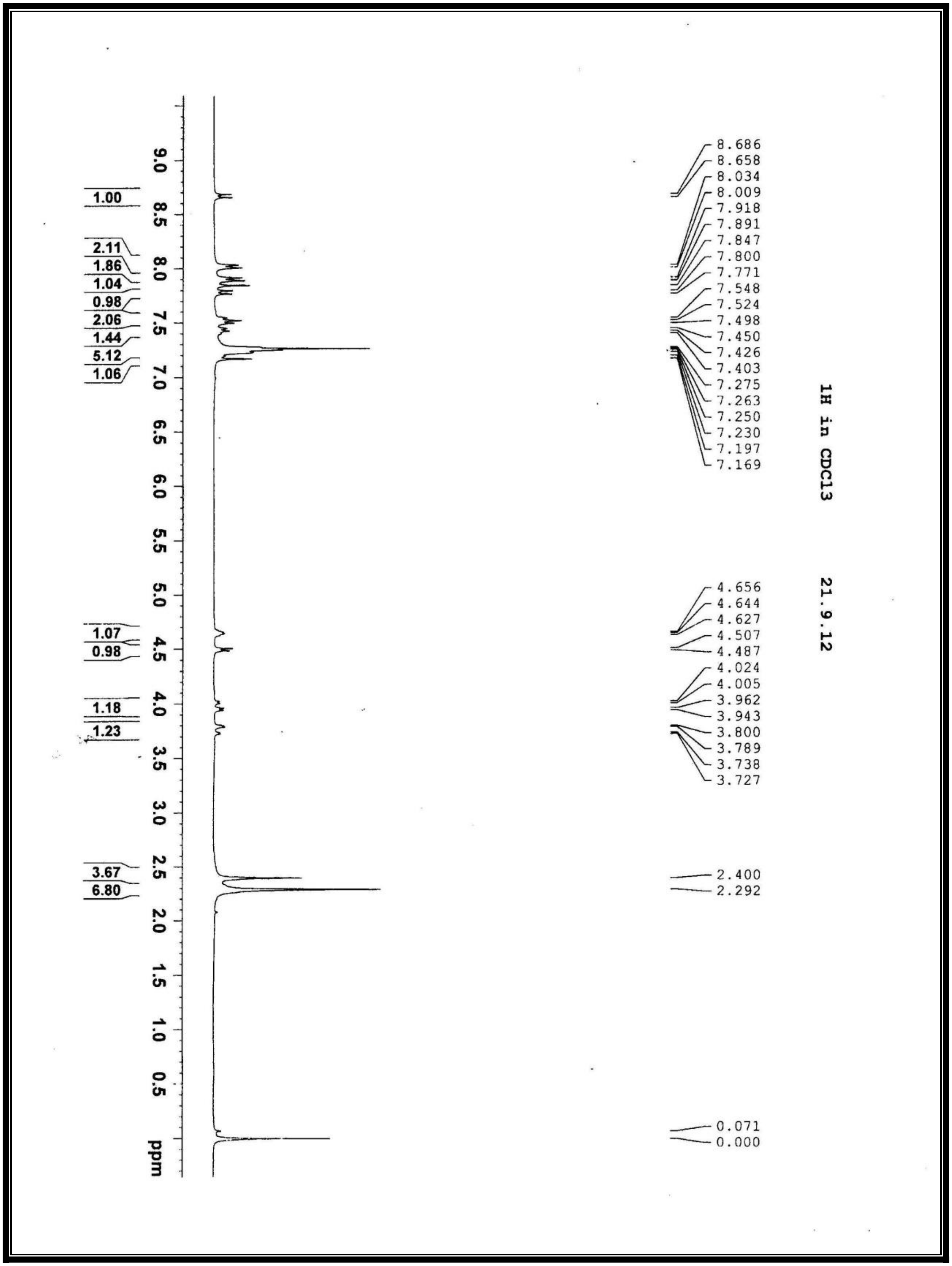

1H in CDCl3
21.9.12
8.686
8.658
8.034
8.009
7.918
7.891
7.847
7.800
7.771
7.548
7.524
7.498
7.450
7.426
7.403
7.275
7.263
7.250
7.230
7.197
7.169
4.656
4.644
4.627
4.507
4.487
4.024
4.005
3.962
3.943
3.800
3.789
3.738
3.727
2.400
2.292
0.071
0.000
1.00
2.11
1.86
1.04
0.98
2.06
1.44
5.12
1.06
1.07
0.98
1.18
1.23
3.67
6.80
ppm

Extension of ^{1}H NMR of **10d**:

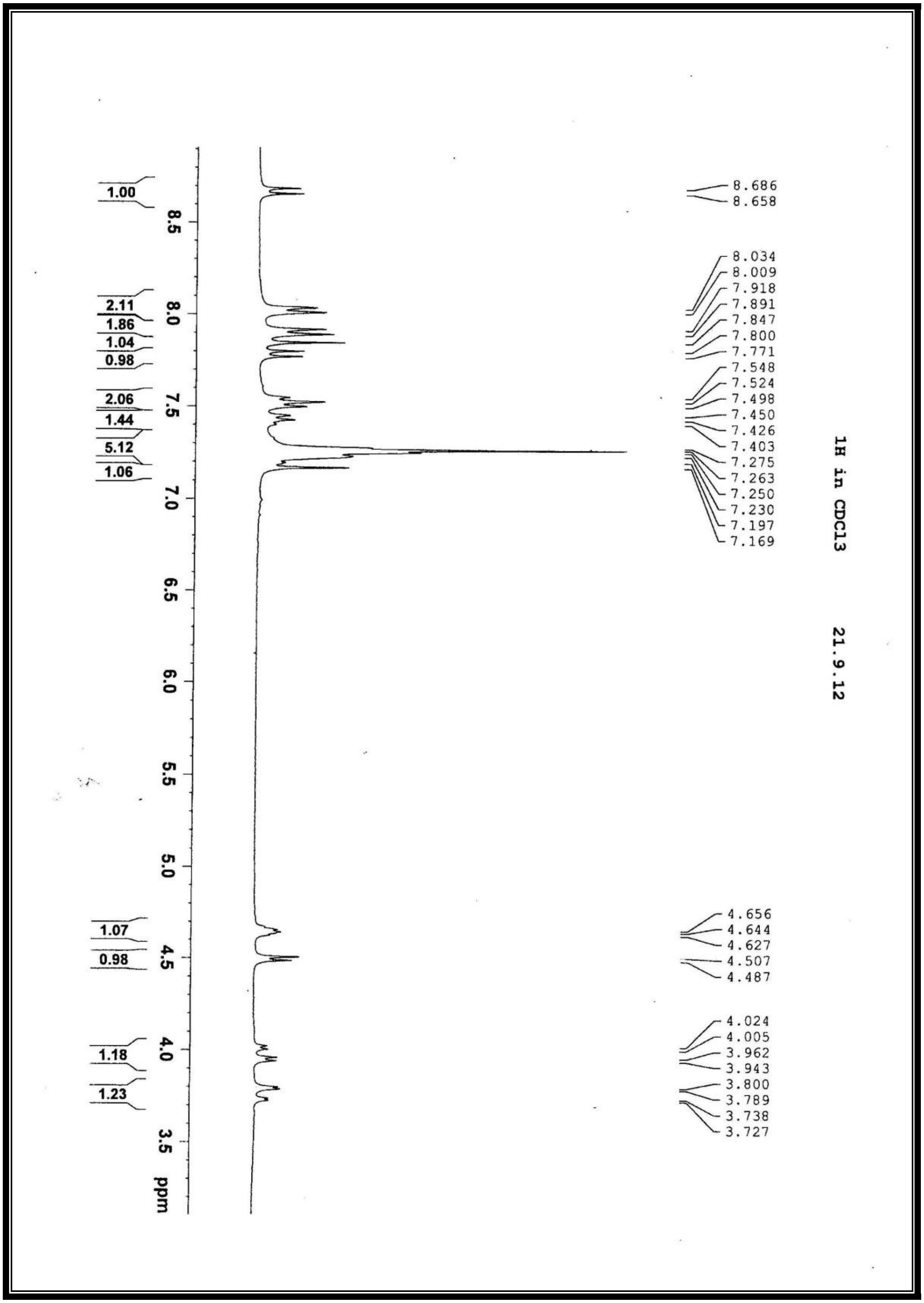

^{13}C NMR of **10d**:

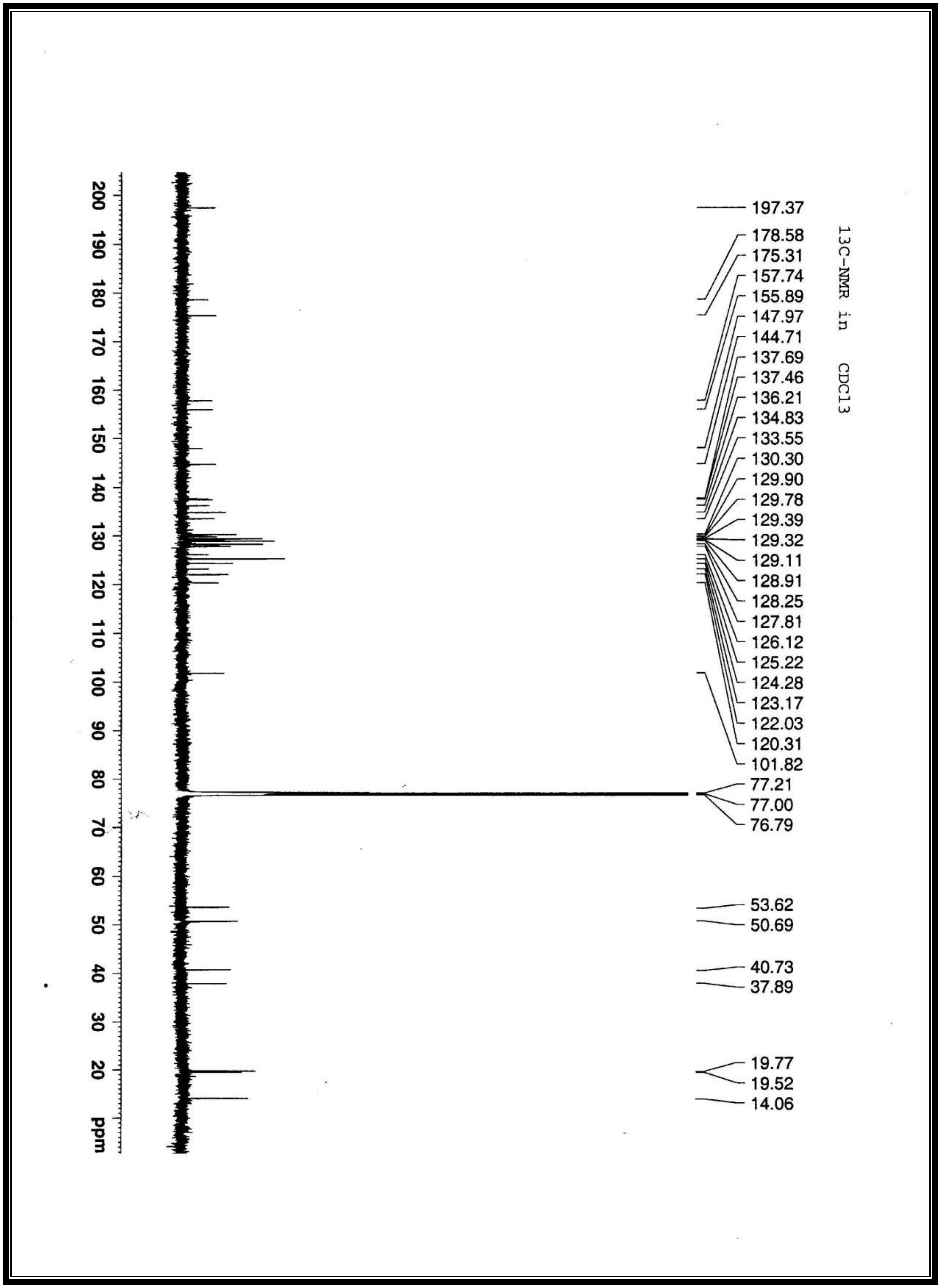

^{1}H NMR of **10e**:

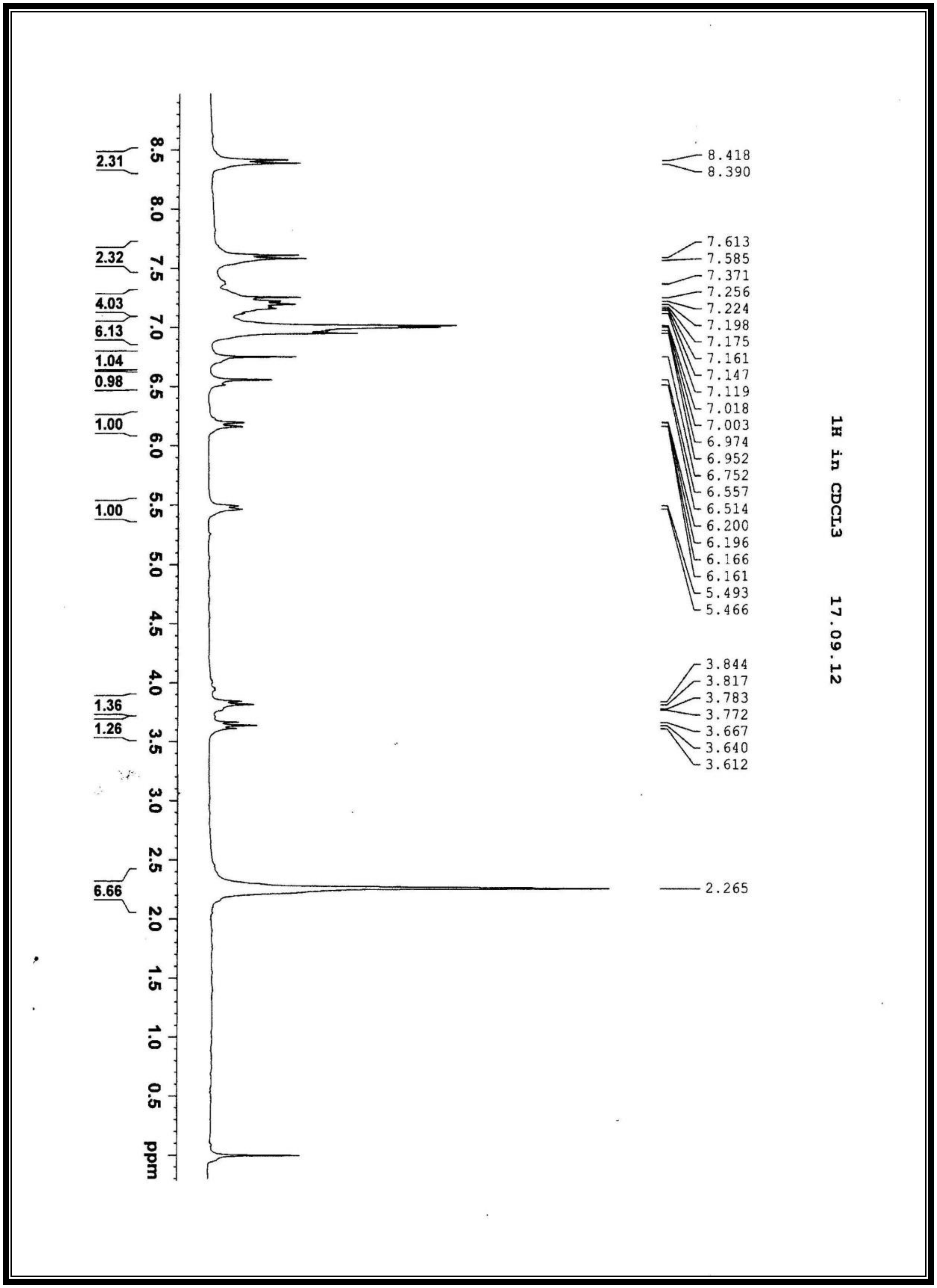

^{13}C NMR of **10e**:

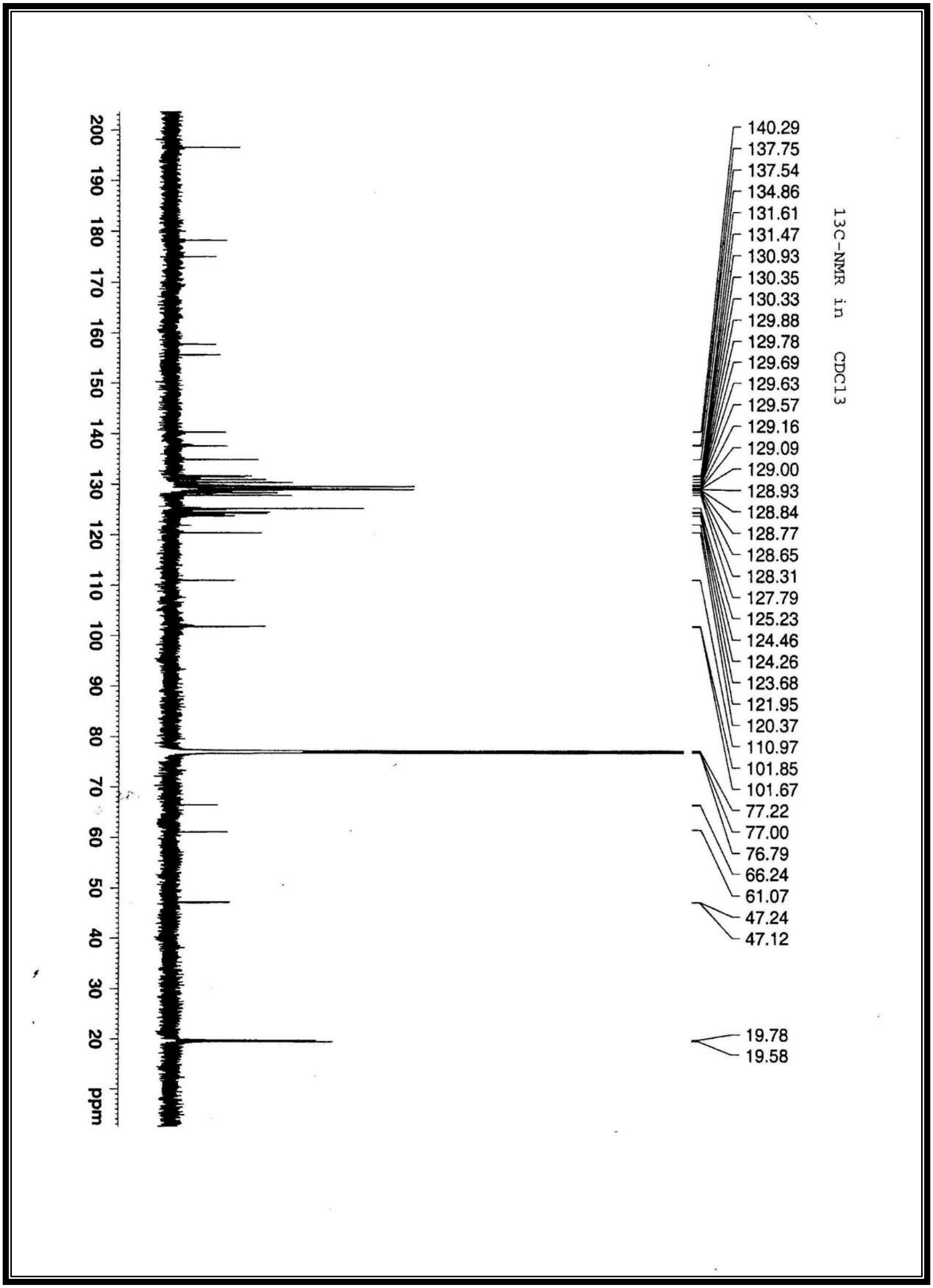

^{1}H NMR of **10f**:

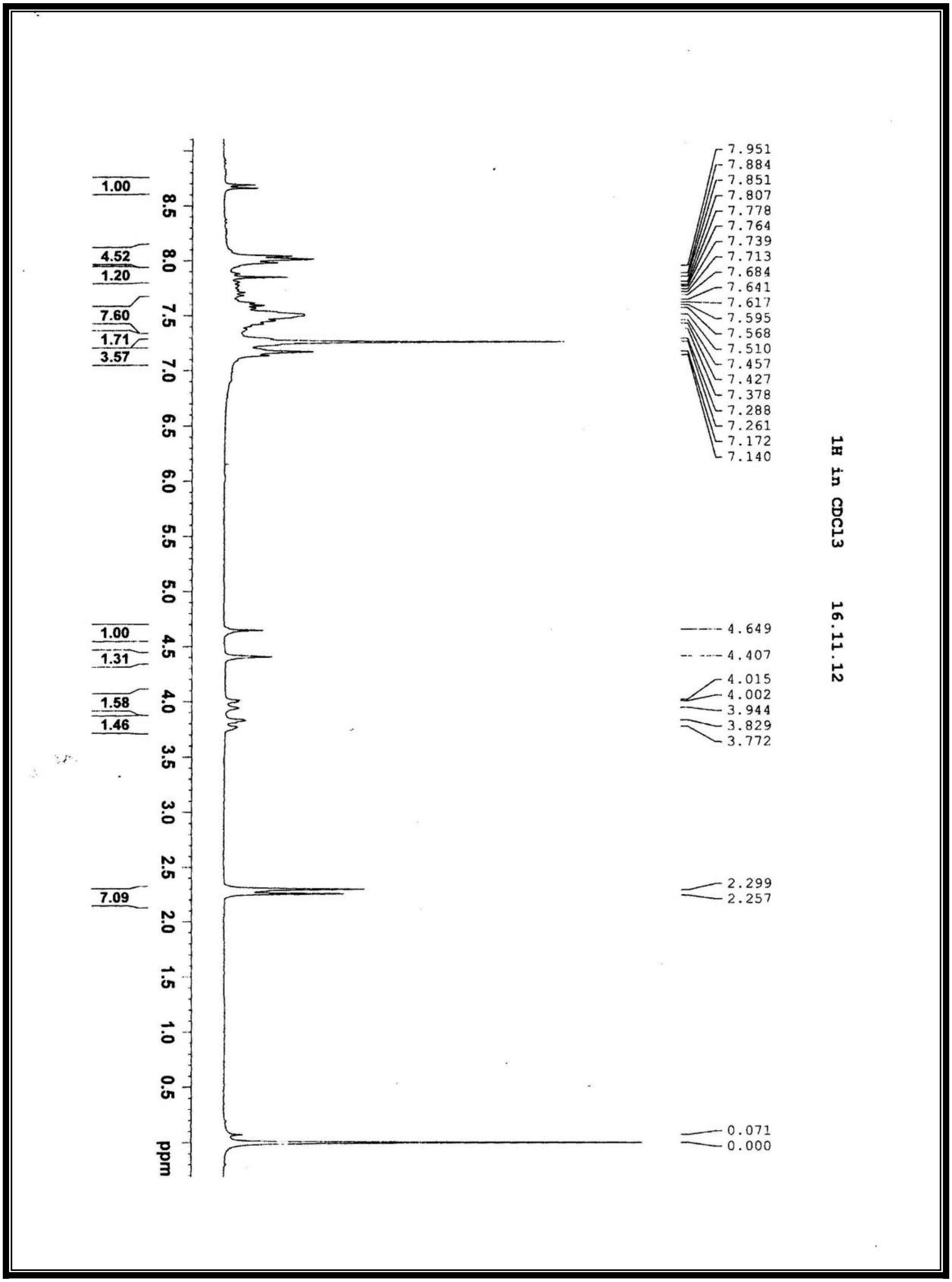

^{1}H NMR of **10g**:

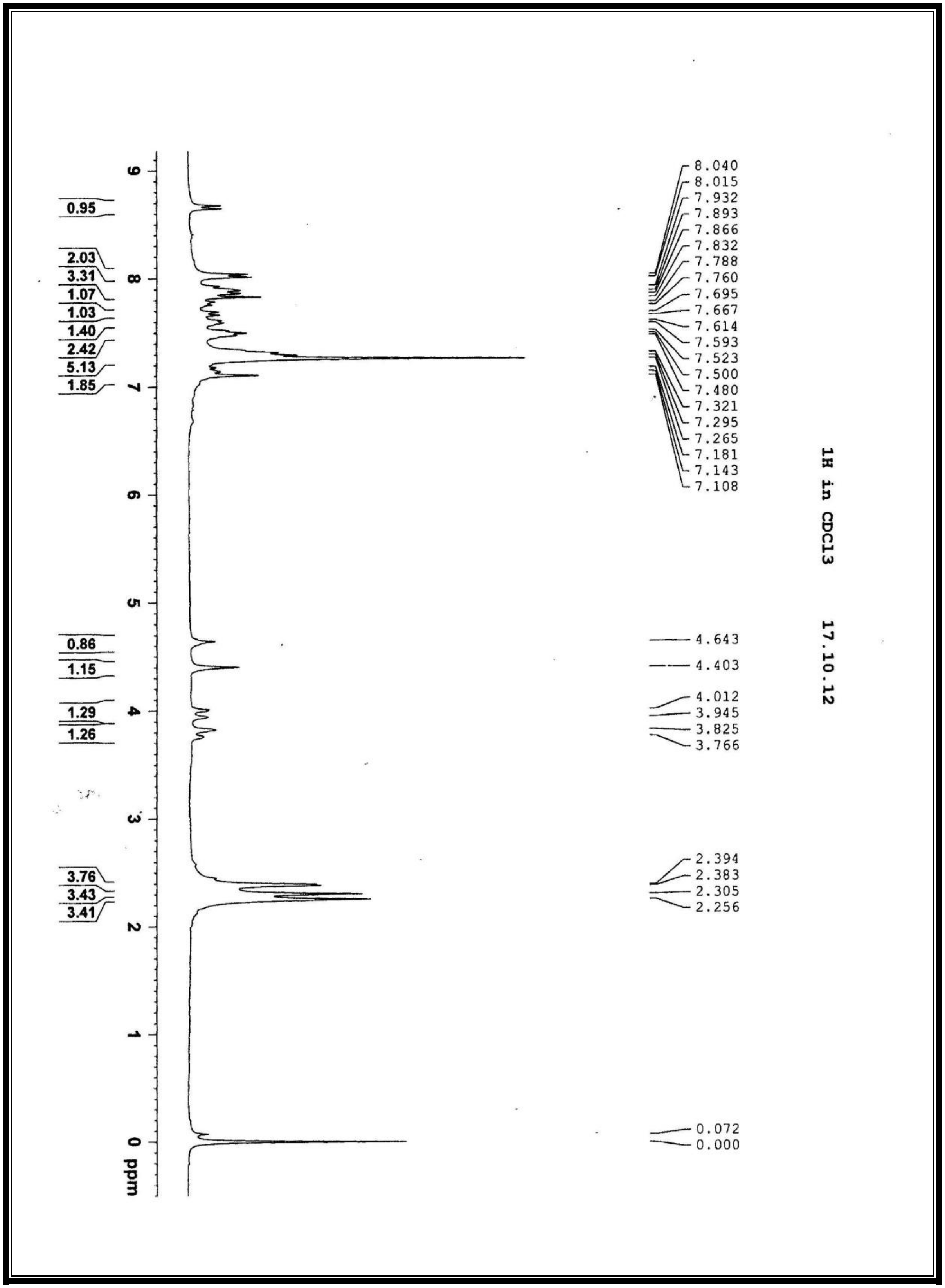
1H in CDCl3 17.10.12
8.040
8.015
7.932
7.893
7.866
7.832
7.788
7.760
7.695
7.667
7.614
7.593
7.523
7.500
7.480
7.321
7.295
7.265
7.181
7.143
7.108
4.643
4.403
4.012
3.945
3.825
3.766
2.394
2.383
2.305
2.256
0.072
0.000
0.95
2.03
3.31
1.07
1.03
1.40
2.42
5.13
1.85
0.86
1.15
1.29
1.26
3.76
3.43
3.41
ppm

^{1}H NMR of **10h**:

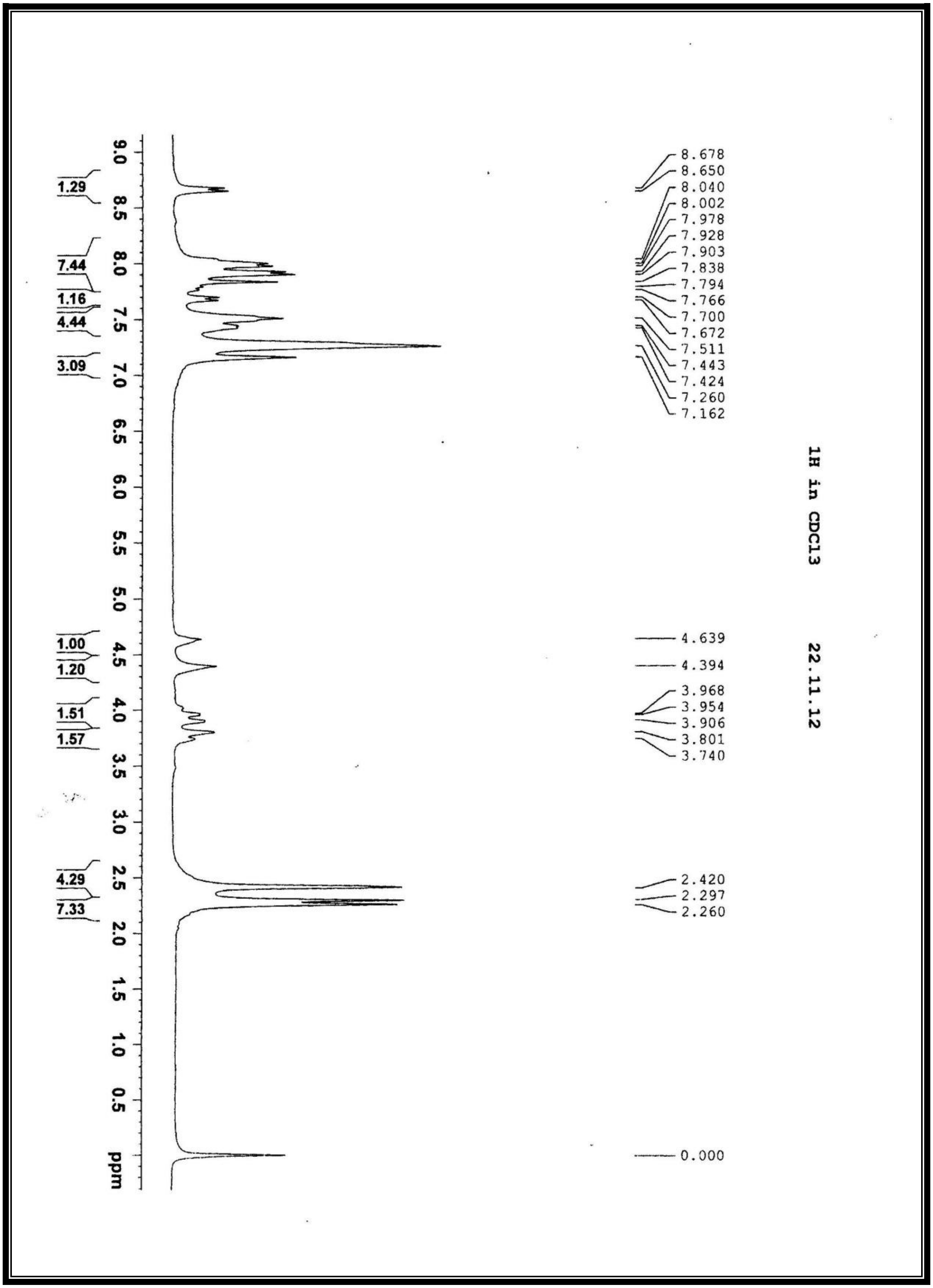
1H in CDCl3
22.11.12
8.678
8.650
8.040
8.002
7.978
7.928
7.903
7.838
7.794
7.766
7.700
7.672
7.511
7.443
7.424
7.260
7.162
4.639
4.394
3.968
3.954
3.906
3.801
3.740
2.420
2.297
2.260
0.000
1.29
7.44
1.16
4.44
3.09
1.00
1.20
1.51
1.57
4.29
7.33
ppm

^{13}C NMR of **10h**:

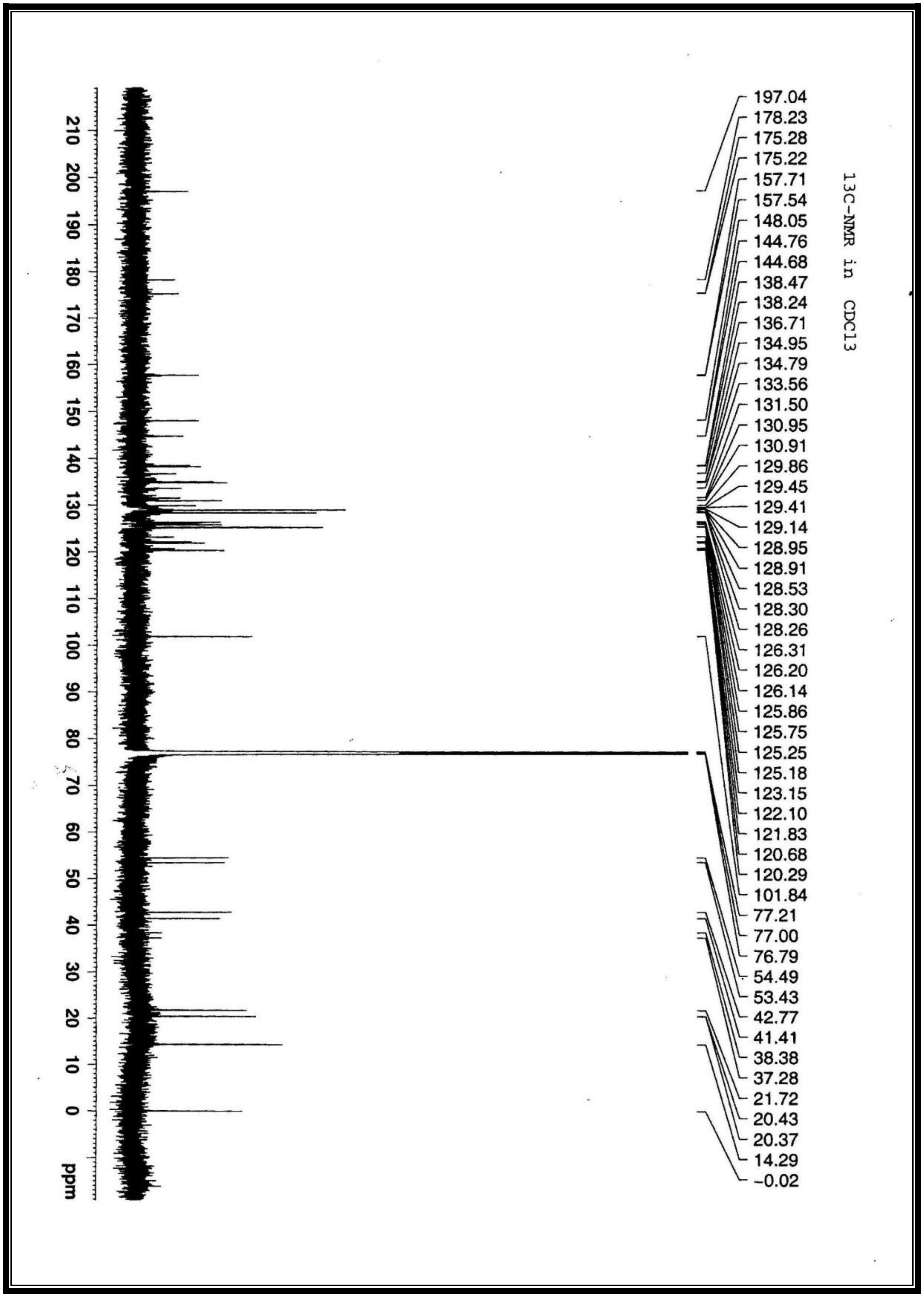

^{1}H NMR of **14a**:

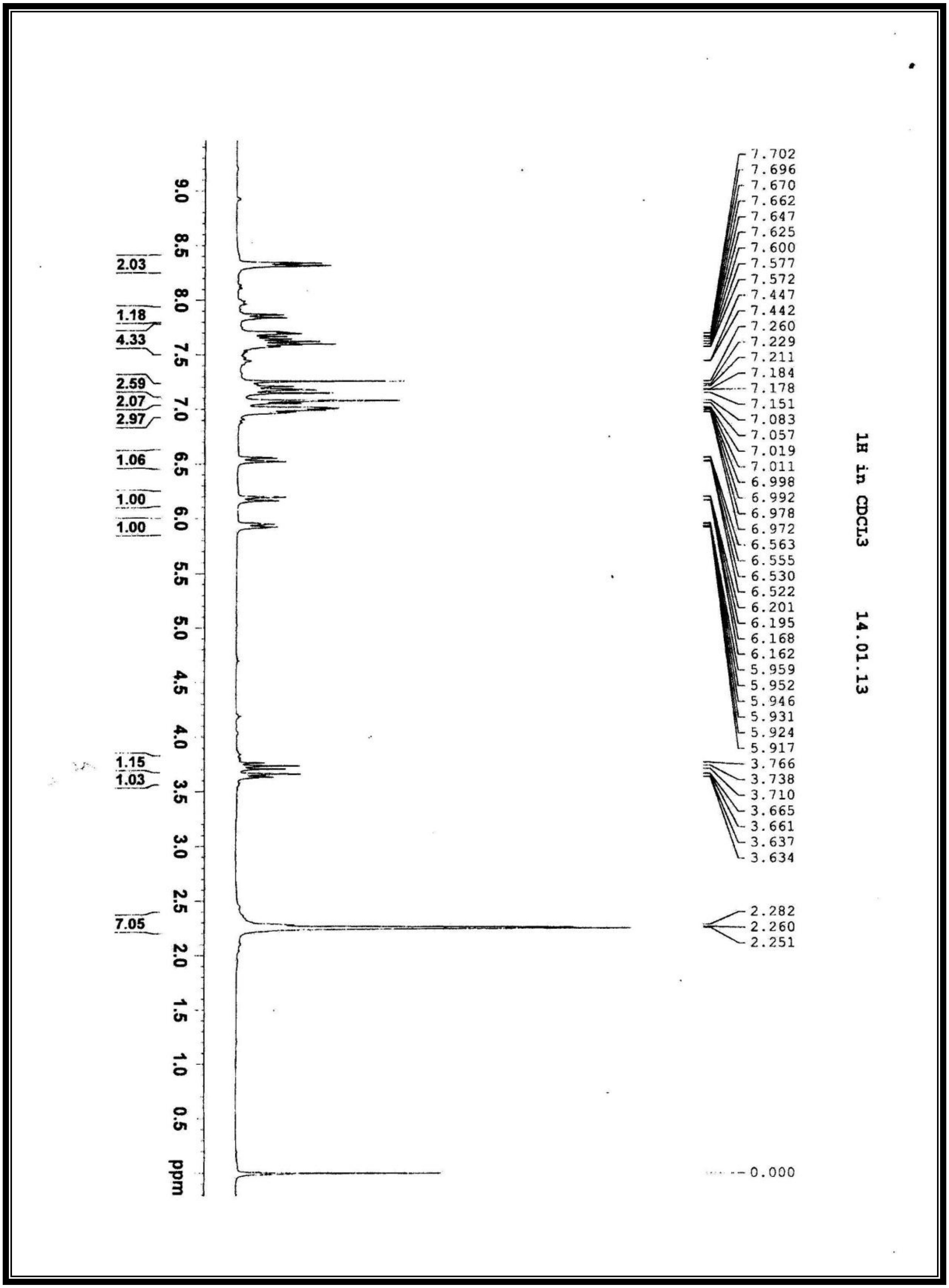
1H in CDCl3 14.01.13
7.702
7.696
7.670
7.662
7.647
7.625
7.600
7.577
7.572
7.447
7.442
7.260
7.229
7.211
7.184
7.178
7.151
7.083
7.057
7.019
7.011
6.998
6.992
6.978
6.972
6.563
6.555
6.530
6.522
6.201
6.195
6.168
6.162
5.959
5.952
5.946
5.931
5.924
5.917
3.766
3.738
3.710
3.665
3.661
3.637
3.634
2.282
2.260
2.251
0.000
2.03
1.18
4.33
2.59
2.07
2.97
1.06
1.00
1.00
1.15
1.03
7.05
9.0
8.5
8.0
7.5
7.0
6.5
6.0
5.5
5.0
4.5
4.0
3.5
3.0
2.5
2.0
1.5
1.0
0.5
ppm

^{13}C NMR of **14a**:

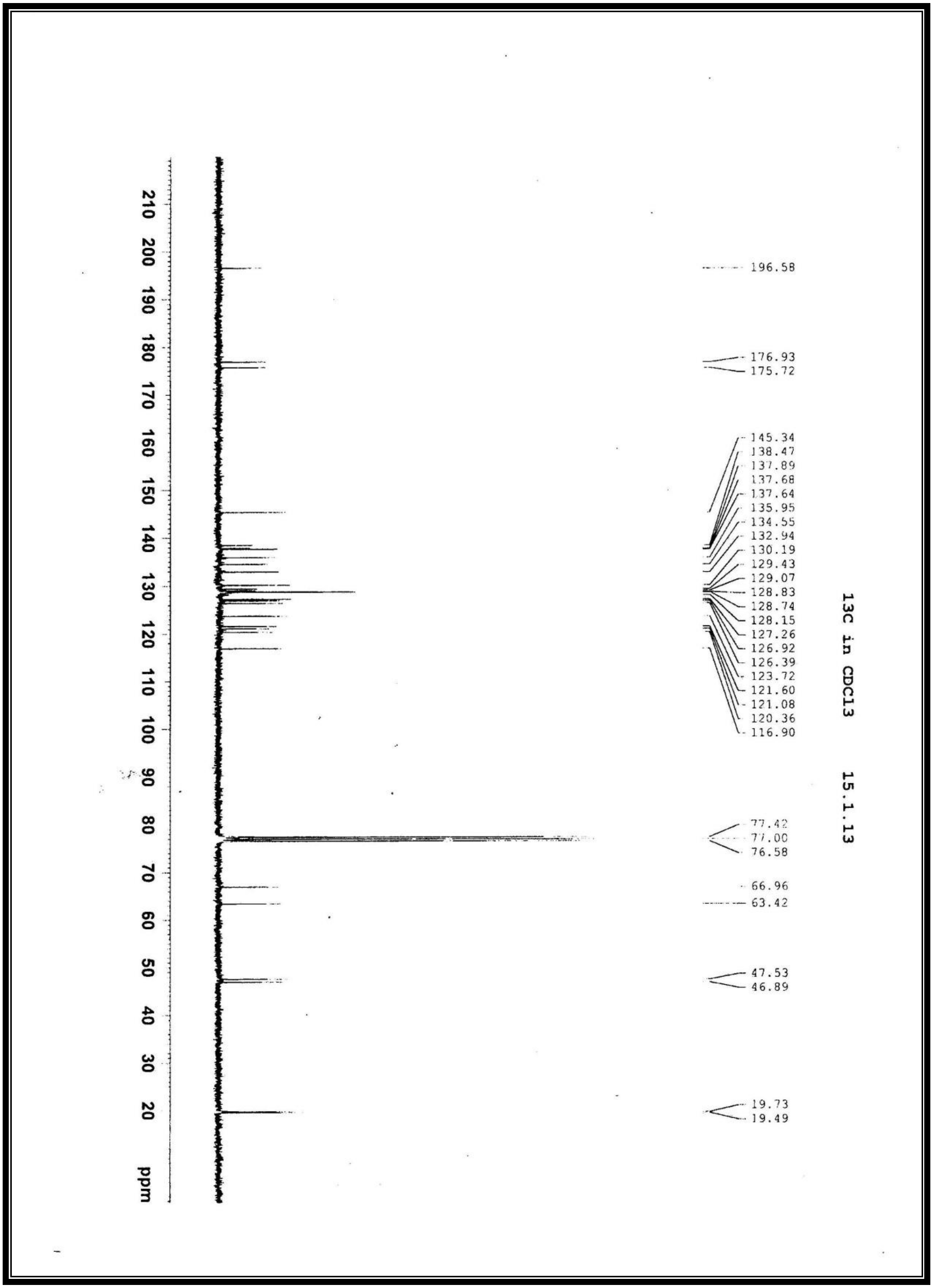

^{1}H NMR of **14b**:

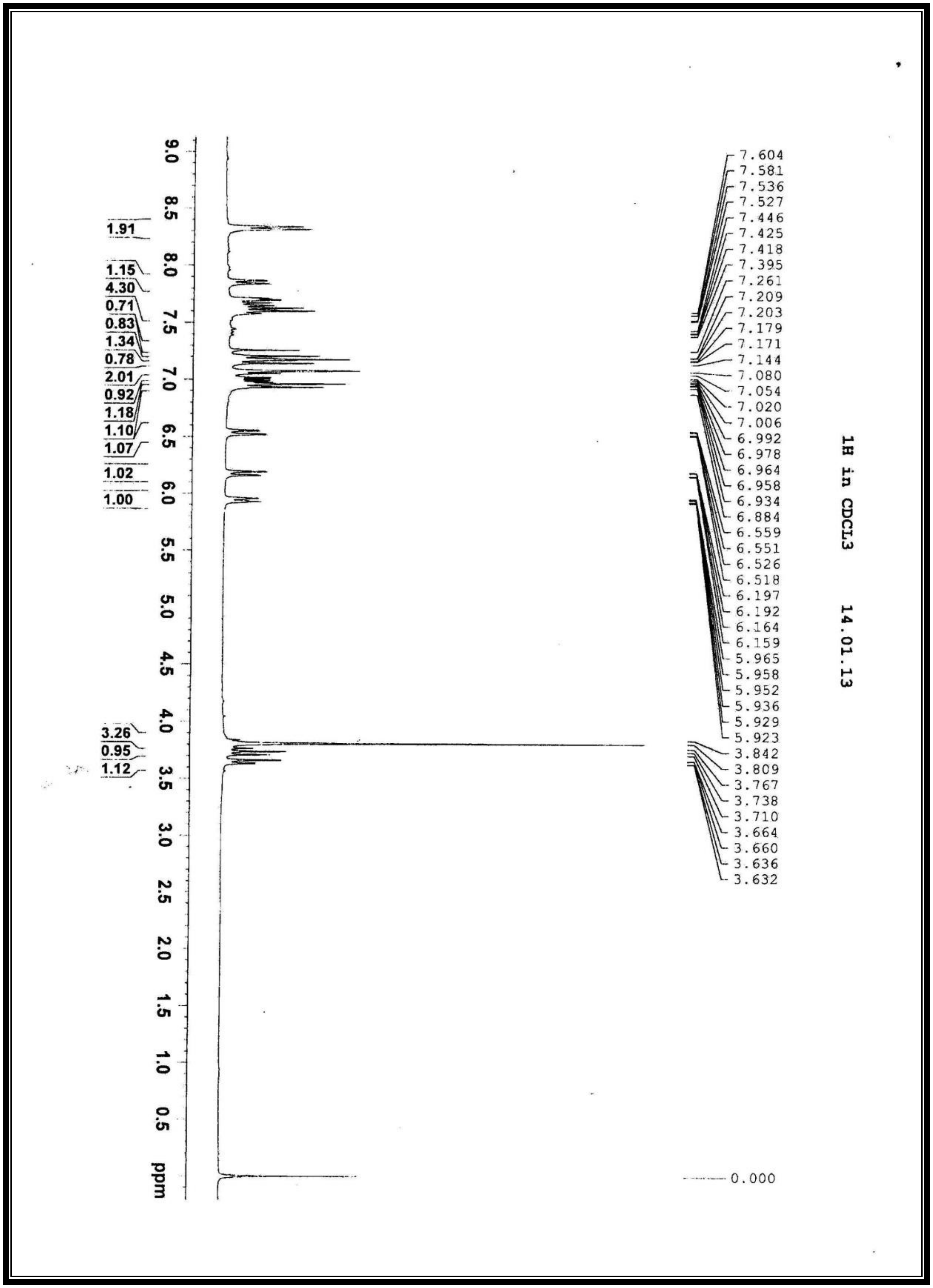

^{13}C NMR of **14b**:

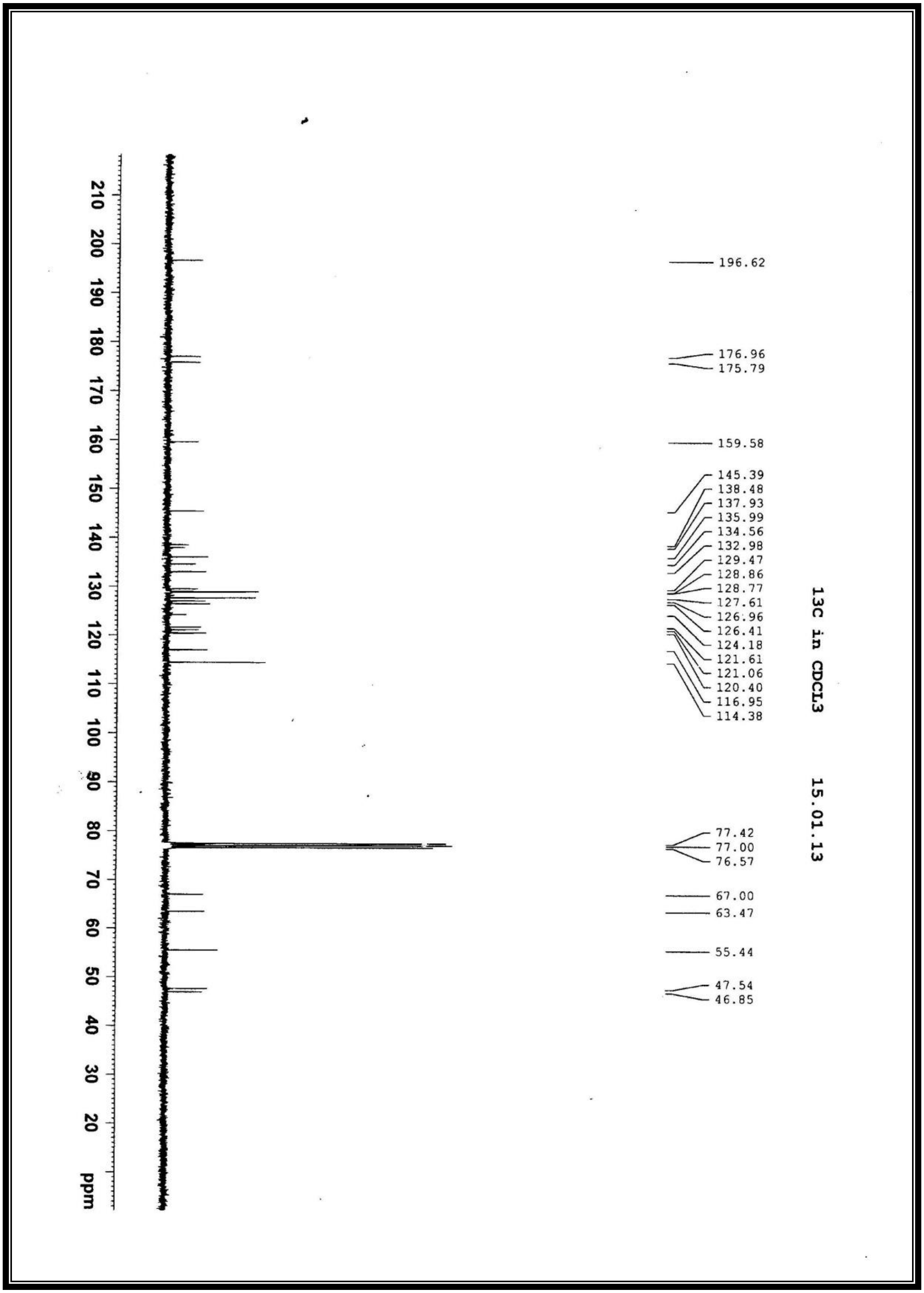

^{1}H NMR of **14c**:

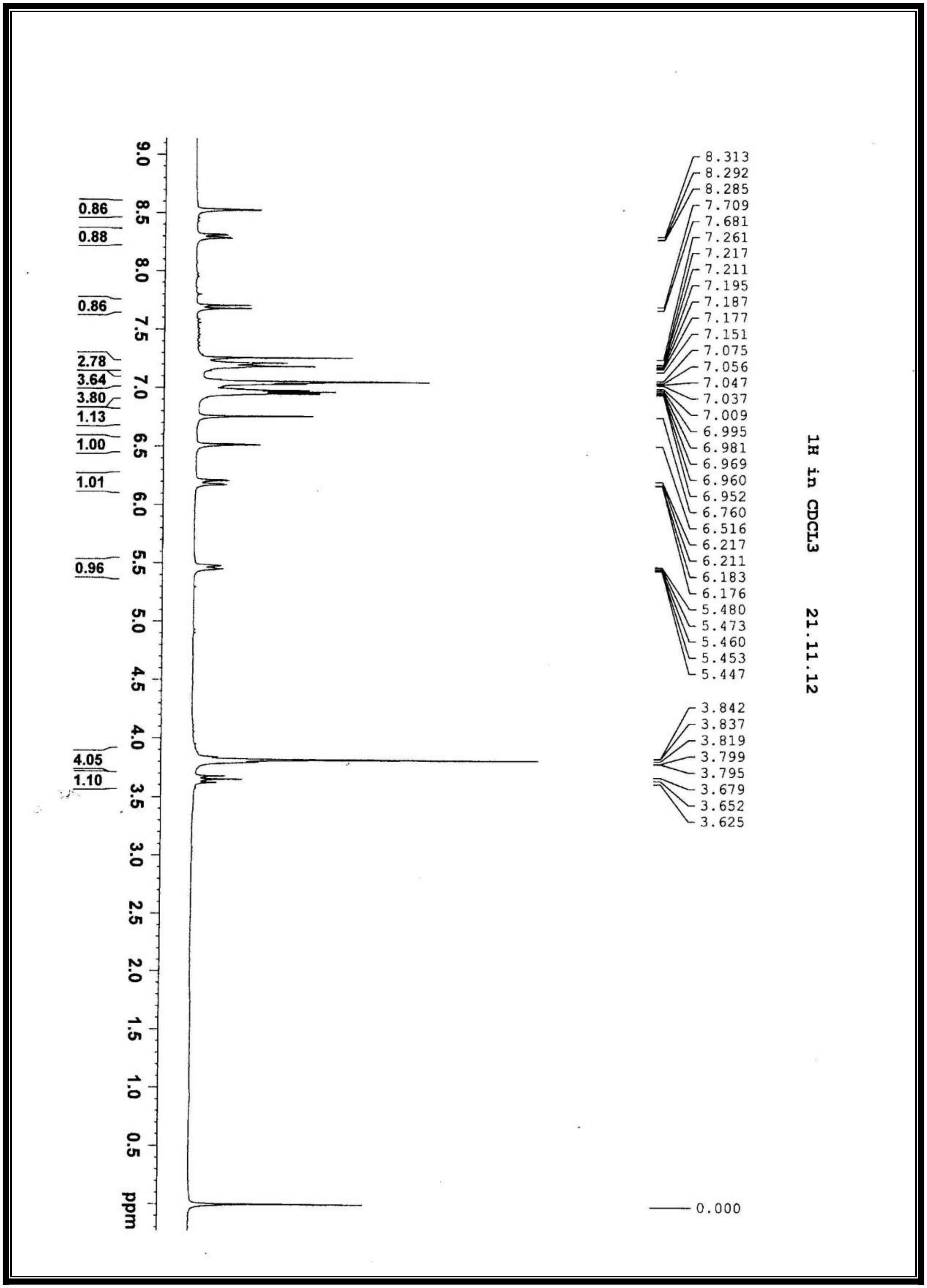
1H in CDCL3 21.11.12
ppm
9.0
8.5
8.0
7.5
7.0
6.5
6.0
5.5
5.0
4.5
4.0
3.5
3.0
2.5
2.0
1.5
1.0
0.5
0.86
0.88
0.86
2.78
3.64
3.80
1.13
1.00
1.01
0.96
4.05
1.10
8.313
8.292
8.285
7.709
7.681
7.261
7.217
7.211
7.195
7.187
7.177
7.151
7.075
7.056
7.047
7.037
7.009
6.995
6.981
6.969
6.960
6.952
6.760
6.516
6.217
6.211
6.183
6.176
5.480
5.473
5.460
5.453
5.447
3.842
3.837
3.819
3.799
3.795
3.679
3.652
3.625
0.000

^{13}C NMR of **14c**:

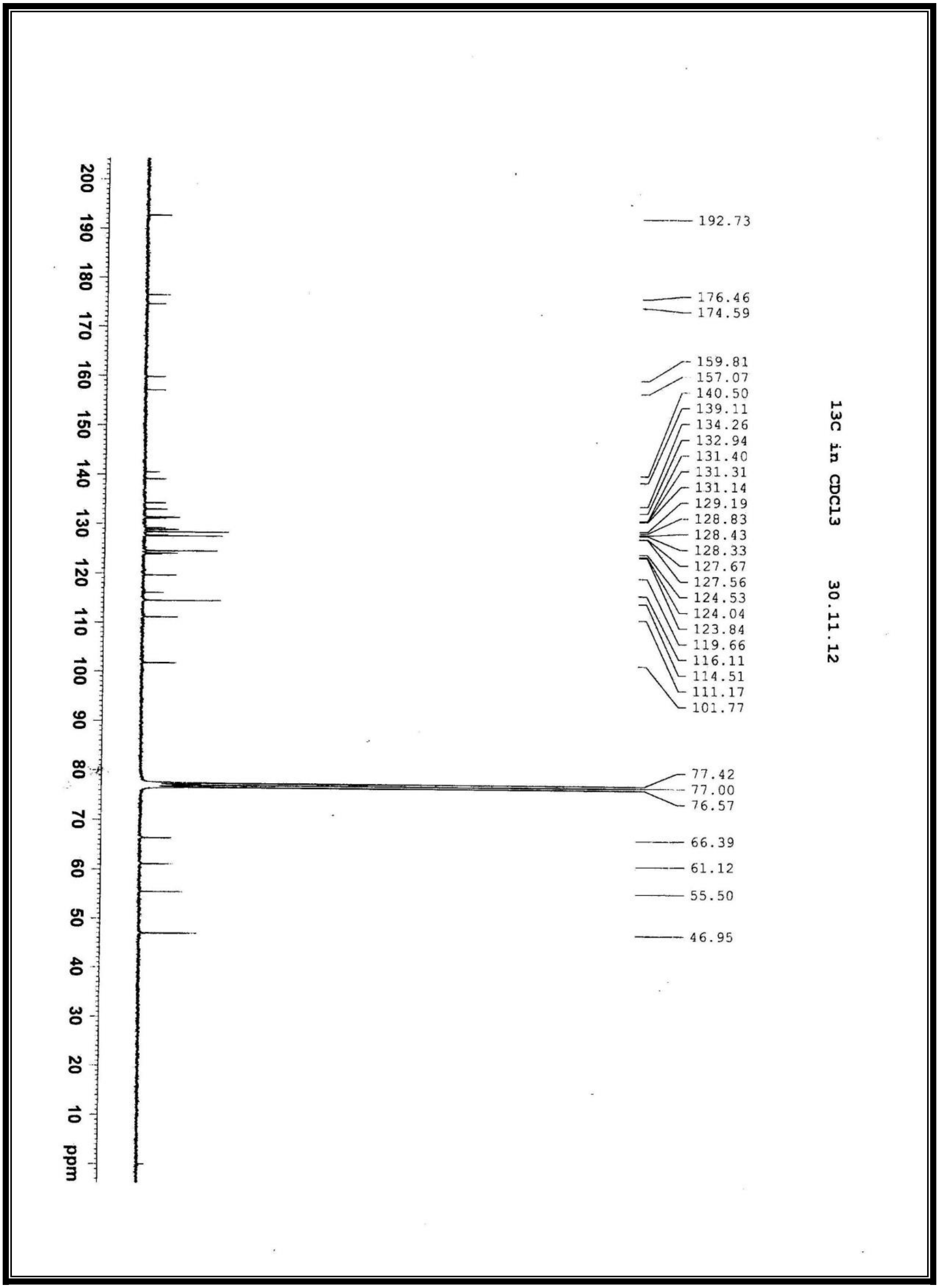

^{1}H NMR of **14d**:

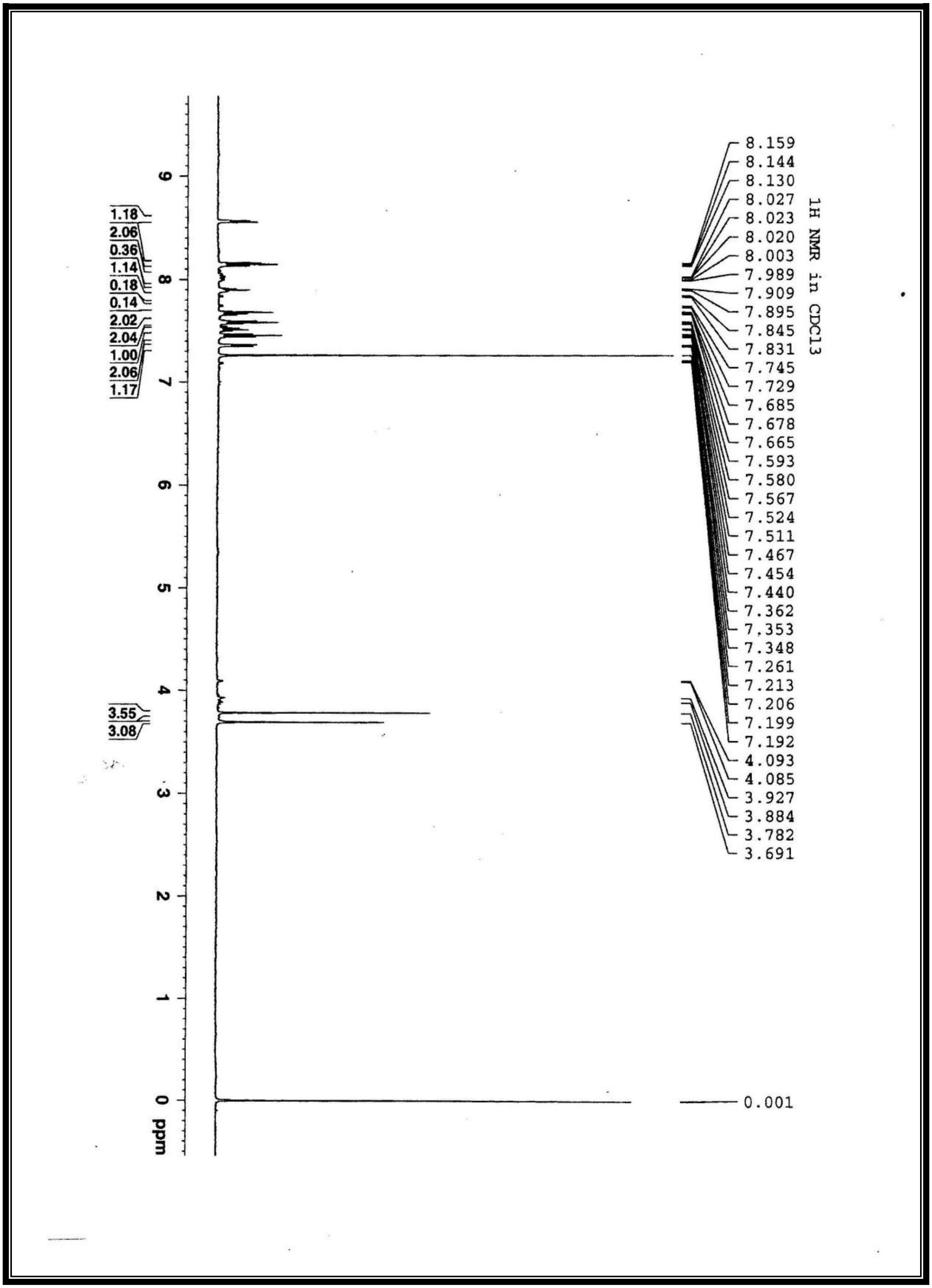

^{13}C NMR of **14d**:

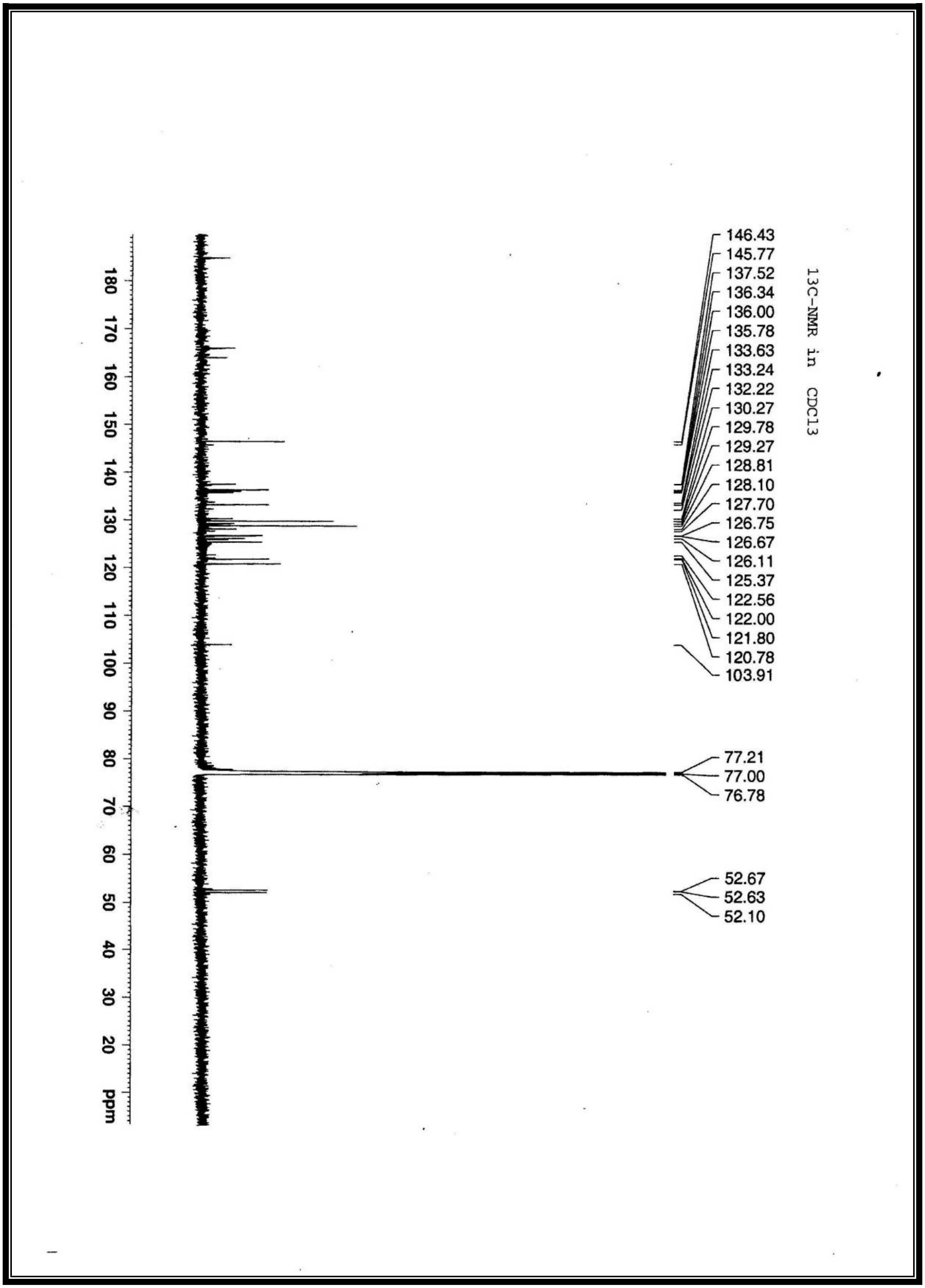

^{1}H NMR of **14e**:

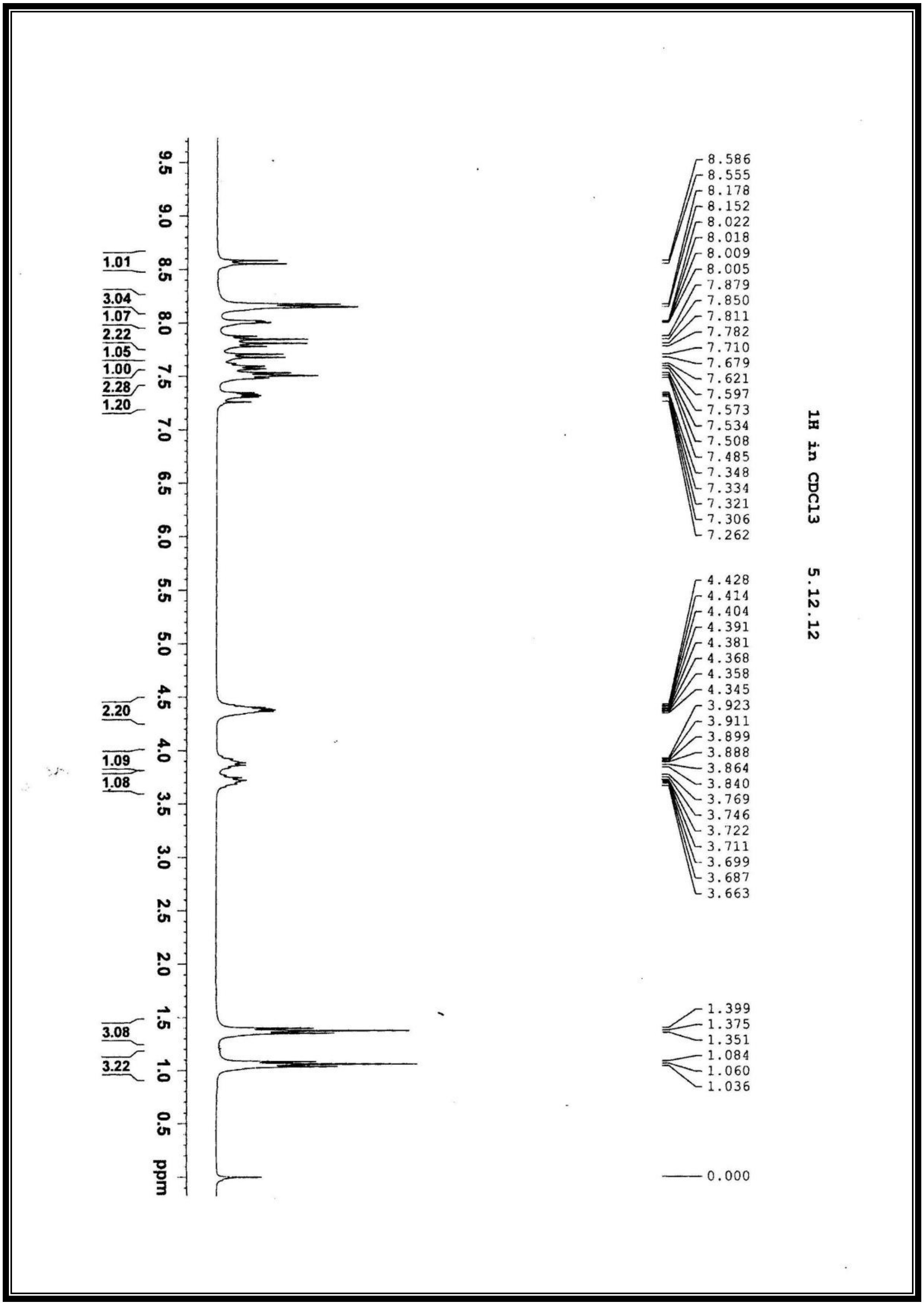
1H in CDCl3 5.12.12
8.586 8.555 8.178 8.152 8.022 8.018 8.009 8.005 7.879 7.850 7.811 7.782 7.710 7.679 7.621 7.597 7.573 7.534 7.508 7.485 7.348 7.334 7.321 7.306 7.262
4.428 4.414 4.404 4.391 4.381 4.368 4.358 4.345 3.923 3.911 3.899 3.888 3.864 3.840 3.769 3.746 3.722 3.711 3.699 3.687 3.663
1.399 1.375 1.351 1.084 1.060 1.036
0.000
1.01 3.04 1.07 2.22 1.05 1.00 2.28 1.20 2.20 1.09 1.08 3.08 3.22
9.5 9.0 8.5 8.0 7.5 7.0 6.5 6.0 5.5 5.0 4.5 4.0 3.5 3.0 2.5 2.0 1.5 1.0 0.5 ppm

^{13}C NMR of **14e**:

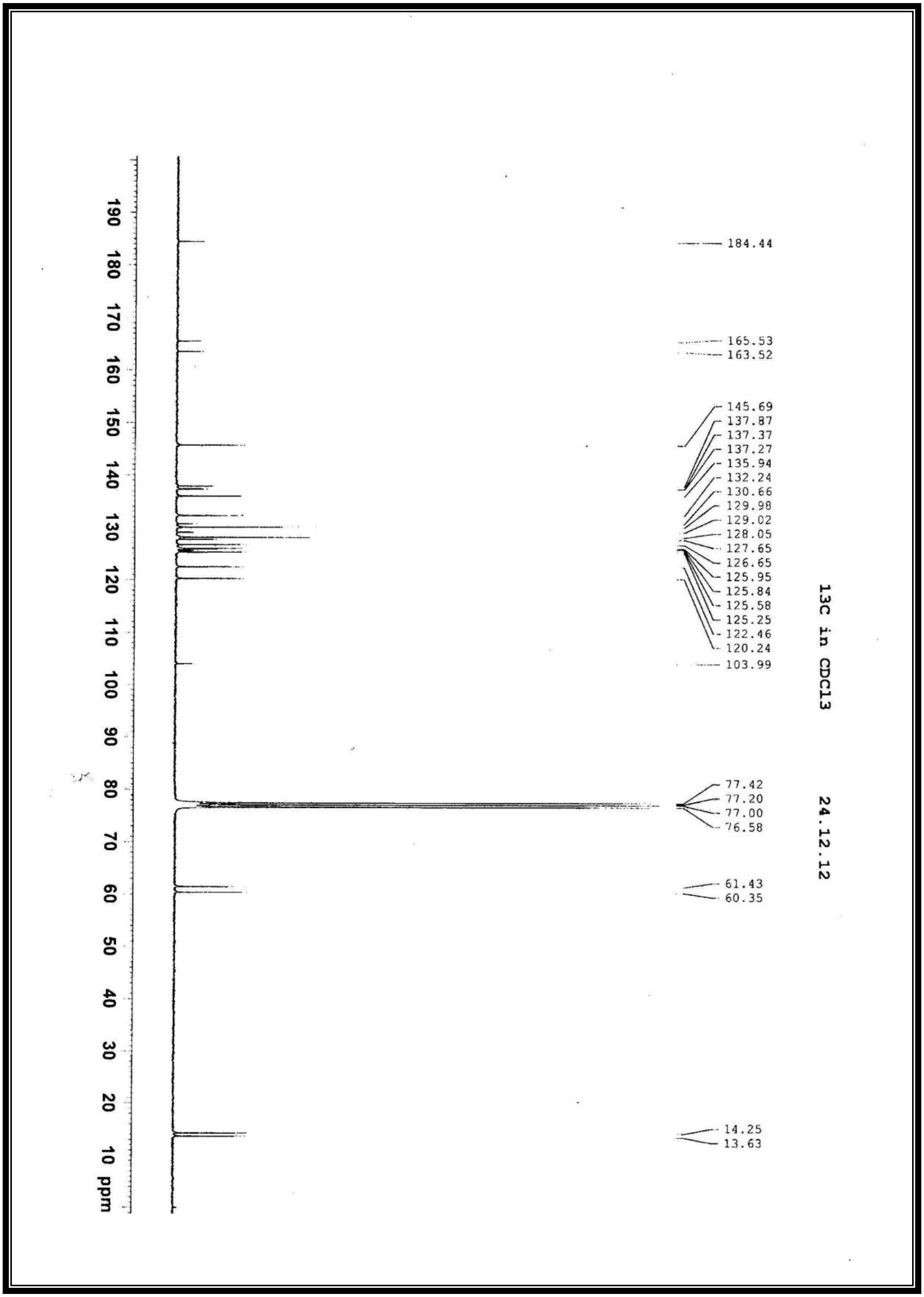

^{1}H NMR of **14f**:

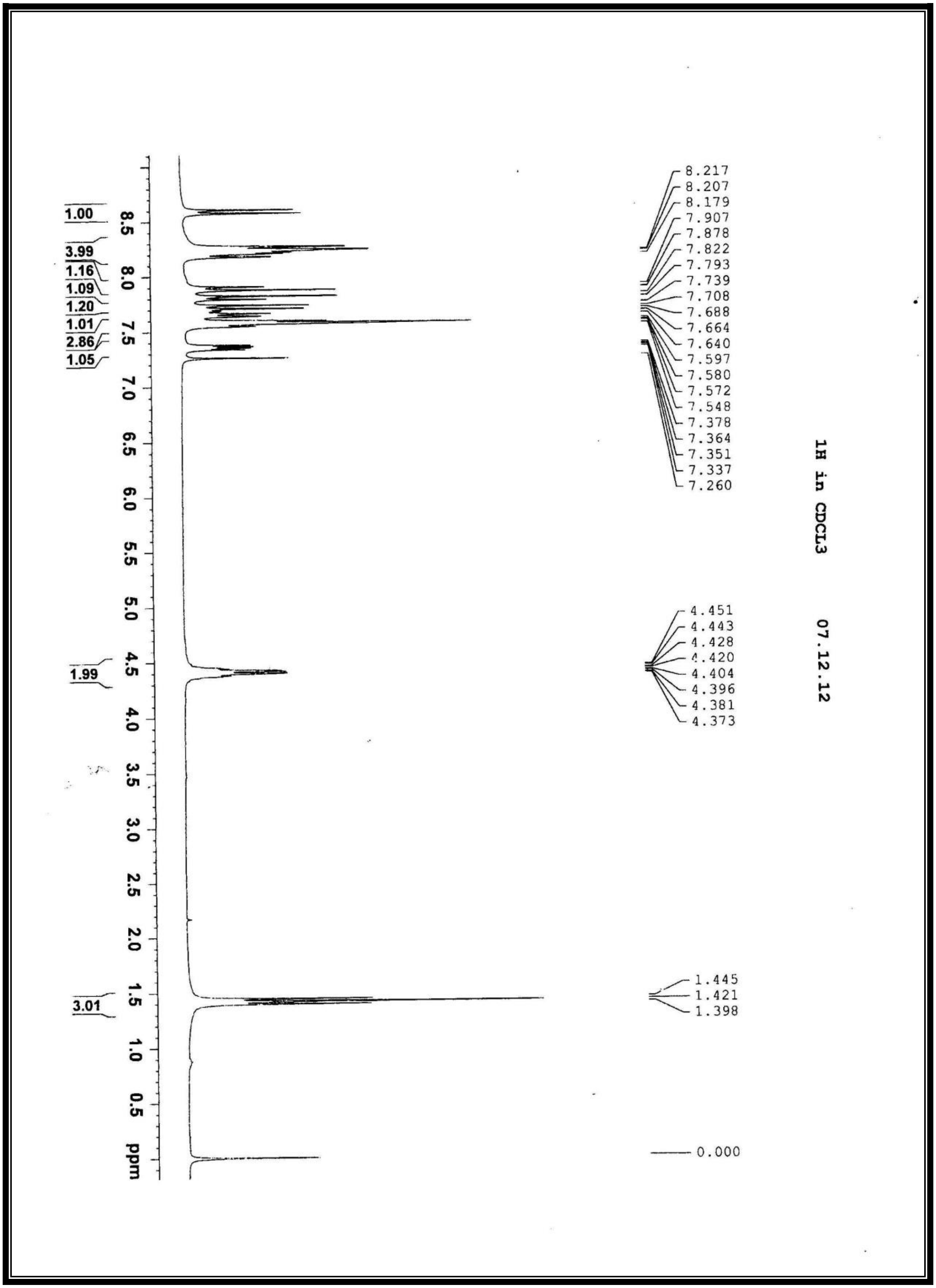
1H in CDCL3
07.12.12
8.217
8.207
8.179
7.907
7.878
7.822
7.793
7.739
7.708
7.688
7.664
7.640
7.597
7.580
7.572
7.548
7.378
7.364
7.351
7.337
7.260
4.451
4.443
4.428
4.420
4.404
4.396
4.381
4.373
1.445
1.421
1.398
0.000
1.00
3.99
1.16
1.09
1.20
1.01
2.86
1.05
1.99
3.01
8.5
8.0
7.5
7.0
6.5
6.0
5.5
5.0
4.5
4.0
3.5
3.0
2.5
2.0
1.5
1.0
0.5
ppm

^{13}C NMR of **14f**:

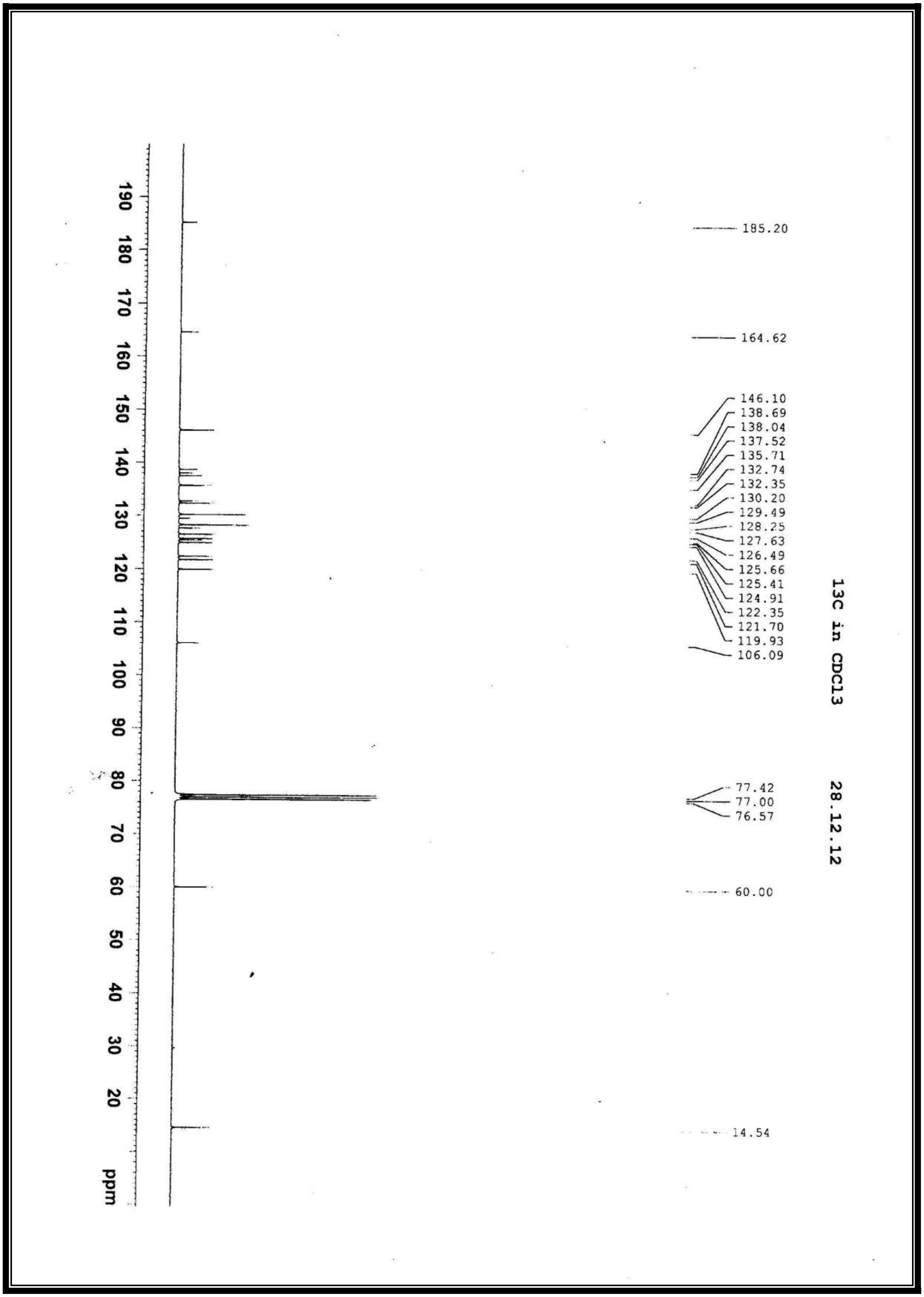

^{1}H NMR of **14g**:

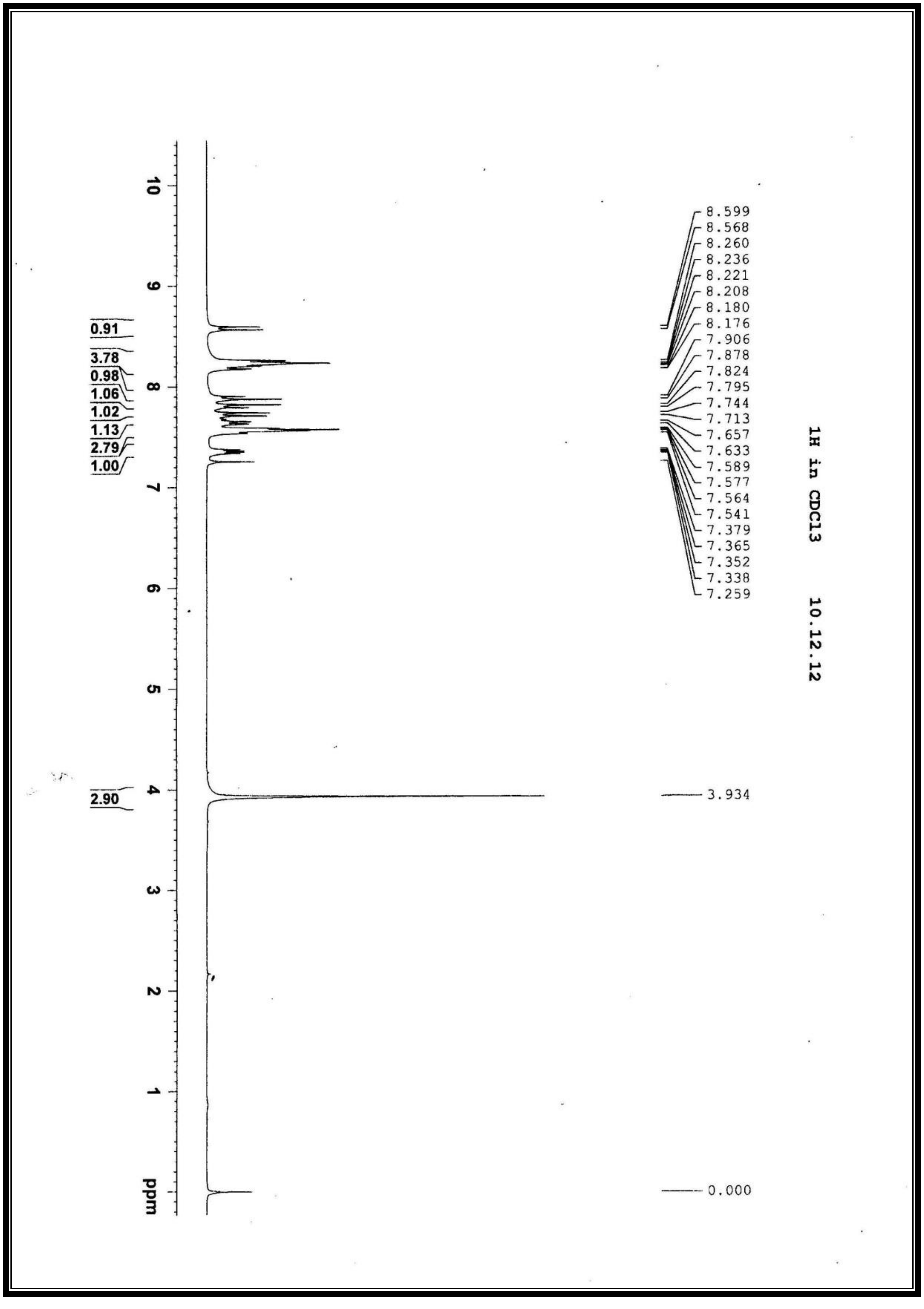

1H in CDCl3 10.12.12
8.599
8.568
8.260
8.236
8.221
8.208
8.180
8.176
7.906
7.878
7.824
7.795
7.744
7.713
7.657
7.633
7.589
7.577
7.564
7.541
7.379
7.365
7.352
7.338
7.259
3.934
0.000
0.91
3.78
0.98
1.06
1.02
1.13
2.79
1.00
2.90
ppm

^{13}C NMR of **14g**:

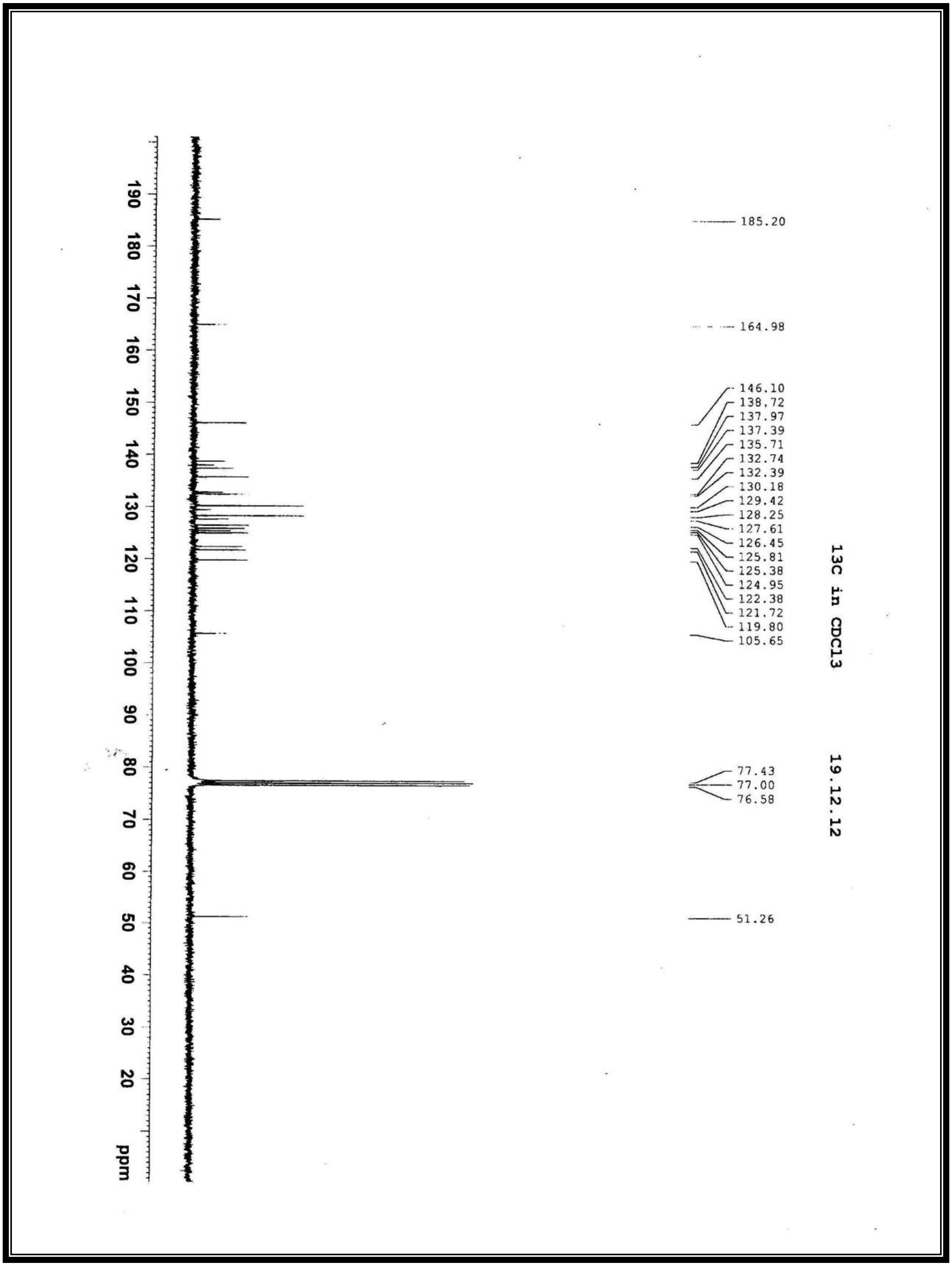

Supplement: File 1 — Experimental and analytical data and copies of 1H NMR and 13C NMR spectra of all new products. [file Beilstein_J_Org_Chem-10-692-s001.pdf]
